# Causal Datasheet for Datasets: An Evaluation Guide for Real-World Data Analysis and Data Collection Design Using Bayesian Networks

**DOI:** 10.3389/frai.2021.612551

**Published:** 2021-04-14

**Authors:** Bradley Butcher, Vincent S. Huang, Christopher Robinson, Jeremy Reffin, Sema K. Sgaier, Grace Charles, Novi Quadrianto

**Affiliations:** ^1^ Department of Informatics, Predictive Analytics Lab (PAL), University of Sussex, Brighton, United Kingdom; ^2^ Surgo Ventures, Washington, DC, United States; ^3^ Harvard T. H. Chan School of Public Health, Cambridge, MA, United States; ^4^ Department of Global Health, University of Washington, Seattle, WA, United States

**Keywords:** bayesian network, causality, causal modeling, lower middle income country, machine learning, big data

## Abstract

Developing data-driven solutions that address real-world problems requires understanding of these problems’ causes and how their interaction affects the outcome–often with only observational data. Causal Bayesian Networks (BN) have been proposed as a powerful method for discovering and representing the causal relationships from observational data as a Directed Acyclic Graph (DAG). BNs could be especially useful for research in global health in Lower and Middle Income Countries, where there is an increasing abundance of observational data that could be harnessed for policy making, program evaluation, and intervention design. However, BNs have not been widely adopted by global health professionals, and in real-world applications, confidence in the results of BNs generally remains inadequate. This is partially due to the inability to validate against some ground truth, as the true DAG is not available. This is especially problematic if a learned DAG conflicts with pre-existing domain doctrine. Here we conceptualize and demonstrate an idea of a “Causal Datasheet” that could approximate and document BN performance expectations for a given dataset, aiming to provide confidence and sample size requirements to practitioners. To generate results for such a Causal Datasheet, a tool was developed which can generate synthetic Bayesian networks and their associated synthetic datasets to mimic real-world datasets. The results given by well-known structure learning algorithms and a novel implementation of the OrderMCMC method using the Quotient Normalized Maximum Likelihood score were recorded. These results were used to populate the Causal Datasheet, and recommendations could be made dependent on whether expected performance met user-defined thresholds. We present our experience in the creation of Causal Datasheets to aid analysis decisions at different stages of the research process. First, one was deployed to help determine the appropriate sample size of a planned study of sexual and reproductive health in Madhya Pradesh, India. Second, a datasheet was created to estimate the performance of an existing maternal health survey we conducted in Uttar Pradesh, India. Third, we validated generated performance estimates and investigated current limitations on the well-known ALARM dataset. Our experience demonstrates the utility of the Causal Datasheet, which can help global health practitioners gain more confidence when applying BNs.

## 1 Introduction

To meet ambitious global health and development goals in lower and middle income countries (LMICs), policy decisions have been increasingly reliant on data-driven approaches to provide necessary insights. This has spawned numerous programs ranging from specific subjects at the sub-national and national level (e.g., Community Behavior Tracking Survey in Uttar Pradesh, India, and the Social And Living Standards Measurement in Pakistan) to broad health topics with multinational participation (e.g., the Multiple Indicator Cluster Surveys developed by the United Nations Children’s Fund, and the USAID-backed Demographic and Health Survey) (Croft et al., 2018; Khan and Hancioglu, 2019; Huang et al., 2020; Pakistan Bureau of Statistics, 2020). These programs have a mandate to collect and disseminate accurate and population-representative health, nutrition, and population data in the developing world. These surveys allow governments and international agencies to monitor trends across health program areas and set priorities for health policy, interventions, and program funding (Fabic et al., 2012). As a result, there has been an explosion of data being generated that has the potential to be used to not only monitor/evaluate the status quo but to inform health intervention design.

Global health and development problems are often complex. An understanding of these complexities is often needed to get the right intervention to the right person, at the right time and place–also known as a Precision Public Health approach (Desmond-Hellmann, 2016; Khoury et al., 2016; Chowkwanyun et al., 2018). Traditionally, for informing intervention design, randomized controlled trials (RCT) remain the gold standard. However, due to cost, lack of infrastructure, and other practical reasons, RCTs are not always possible in LMICs. As a result, many of the available data collected are observational only and limited in scope. Without an RCT, quantifying which variables are the proximate causes of an outcome or determining causes and effects for specific set of variables remains a challenge for global health practitioners. Moreover, RCTs are by design conducted with the intent to test a narrow set of hypotheses, not to explore unknown causal structures - a potential missed opportunity to target public health solutions more precisely.

Causal inference and discovery approaches such as causal Bayesian Network (BN) can fill this void. BNs readily deal with observational data, can utilize numerous algorithms to facilitate automatic causal discovery, allow for expert-specified constraints, and can infer the causal effects of hypothetical interventions (Pearl, 1995; Arora et al., 2019; Glymour et al., 2019). Despite causal Bayesian Network’s many offerings, we have not seen a wide adoption in real-world problems (Arora et al., 2019; Kyrimi et al., 2020; Sgaier et al., 2020). We have found that validating the structure, parameterization, predictive accuracy, and generalizability of BN presents a significant hurdle and is subject to considerable debate and interpretation when applied to data with real-world complexity. Our inability to communicate uncertainty in structure learning algorithm performance for specific datasets can call entire models into question (van der Bles et al., 2019). Generally, practitioners using BNs must resort to domain expertize to validate model structure, if they do not forgo validation entirely (Aguilera et al., 2011; Lewis and McCormick, 2012; Moglia et al., 2018). This makes BN model results especially difficult to defend when they, even if just in part, contradict previous domain beliefs or doctrines. Thus, BN results are often presented as a proof-of-concept of techniques to show that the method can recover insights already known rather than as an actionable model for discovery, change, or intervention (Lewis and McCormick, 2012; Moglia et al., 2018; Raqeujo-Castro et al., 2018).

The problem of not knowing how well machine learning algorithms will perform in real-world conditions is not restricted to causal discovery and inference and has been subject to a broader debate. One proposed solution is adopting the standard “datasheet” practice of constructing and accompanying any given dataset with a full description of the data, its collection context, operating characteristics (i.e., the characteristics of the data to which a machine learning algorithms is applied), and test results (i.e., the expected performance of the machine learning algorithm) (Gebru et al., 2018). Measuring the expected causal discovery and inference performance and their uncertainties for any given dataset is, however, not straightforward. First, it is not clear what performance metrics should be used to measure BN algorithms’ ability to recover the ground truth causal structure when the ground truth is unknown. In addition, such data may not include the appropriate variables to establish causal or interventional sufficiency, can have incomplete observations, and may be imbalanced (Spirtes et al., 2000; Pearl, 2009; Kleinberg and Hripcsak, 2011; Peters et al., 2017). Lastly, the sample size of a dataset may be insufficient to support BN analyses (Wang and Gelfand, 2002). Perhaps due to the data challenges mentioned above, the evaluation of novel BN algorithms has been largely based on standard synthetic datasets such as ALARM, Insurance, Child and others (Beinlich et al., 1989; Dawid, 1992; Binder et al., 1997; Scutari, 2009), which can have vastly different characteristics compared to real-world data at hand. One suggested method for ranking algorithms’ performance is to assume the intersection of the structures found by a collection of algorithms as the partial ground truth as in the Intersection-Validation method by Viinikka et al. (2018). However, the Intersection-Validation method will often neglect to consider the most complex relationships, and while it provides relative sample size requirements for each algorithm, it cannot directly inform the data collection process. We face the following quandary: with real-world data we lack the ground truth against which to evaluate the modeling algorithms, and with synthetic data we lack the complexity and limitations that are typically imposed in real-world circumstances (Gentzel et al., 2019).

To solve this quandary and to empower practitioners to estimate uncertainty levels around the causal structures learned under the typical contexts and constraints applicable to their analytical problem of interest, we propose an approach to attach two types of causal extension to such datasheet proposed by Gebru et al. (2018) to 1) inform study design at the data collection stage to enable subsequent causal discovery analysis similar to, in spirit, conducting power analysis before sample size is determined, and 2) describe expected causal discovery and inference algorithm performance and corresponding uncertainty when presented an existing dataset. The key idea is to generate synthetic data with a spectrum of properties that mimic the existing or projected real-world data. We call our instantiation of this capability the ‘Causal Datasheet Generation Tool’, or CDG-T.

In this work, our goal is to provide further confidence in BN results from the perspective of practitioners’ needs. BNs are introduced in 2.1 of the Materials and Methods Section. In Section 2.2 we briefly look at pertinent related work. In Section 2.3, we introduce the approach taken in generating causal datasheets, including a brief discussion the assumptions that are made. Following this in Sections 2.4–2.7 we define the data characteristics used to generate synthetic data, what structure learning algorithms were explored, definitions of the performance metrics used in the datasheets, and the two datasheet usage scenarios. In Section 3, Results, we illustrate the usage of three example datasheets. First, to inform data collection design in an LMIC setting, we provide an example on how a Causal Datasheet was used in planning of a Sexual Reproductive Health survey in Madhya Pradesh, India, where the performance value is computed over a range of potential variables and sample sizes. Next, for evaluating data suitability for BN we provide two example Causal Datasheets for existing data evaluation: one example for an existing dataset in the global development domain (a survey about Reproductive Maternal Neonatal Child Health (RMNCH) that we administered in Uttar Pradesh, India), and another generated for the well-known ALARM dataset (Beinlich et al., 1989). Lastly, we note the implications and future research directions in the Discussion.

## 2 Materials and Methods

### 2.1 Causal Bayesian Network

**FIGURE 1:**
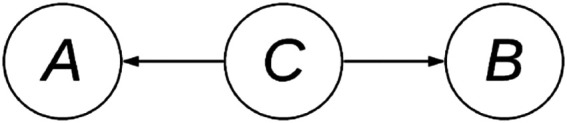
A DAG with three variables **(A–C)** and two edges.

A Bayesian network (G,Θ) for a set of variables *X* consists of two components: a directed acyclic graph (DAG), and a set of parameters Θ. The DAG (V,E) of a BN encodes the statistical dependence among the set of variables *X* by means of the set of edges *E* which connect nodes *V* (Figure 1). Each node $V_{i}\in V$ corresponds to one variable $X_{i}\in X$.

Conversely, the absence of an edge between variables suggests a statistical (conditional) independence. Thus, a BN induces the factorization:

$$P\left(X|G,\text{Θ}\right)=\overset{D}{\underset{i=1}{\prod }}P\left(X_{i}|{\text{Π}}_{X_{i}},{\text{Θ}}_{X_{i}}\right)$$

where the global distribution P(X|G,Θ) factorizes into a set of local distributions; one for each $X_{i}$ with parameters ${\text{Θ}}_{X_{i}}$, conditional on its parents ${\text{Π}}_{X_{i}}$.

Discrete BNs assume that a variable $X_{i}$ is distributed multinomially conditioned on a configuration of its parents $\left(X_{i}=k|{\text{Π}}_{X_{i}}\right)∼Mul\left(\pi_{ik|j}\right)$, where $\pi_{ik|j}=\text{P}\left(X_{i}=k|{\text{Π}}_{X_{i}}=j\right)$ is the probability when $X_{i}=k$ conditioned on the *j*th value of the possible parent combinations. These discrete conditional distributions can be represented as conditional probability tables (CPTs) (Heckerman et al., 1995).

**FIGURE 2:**
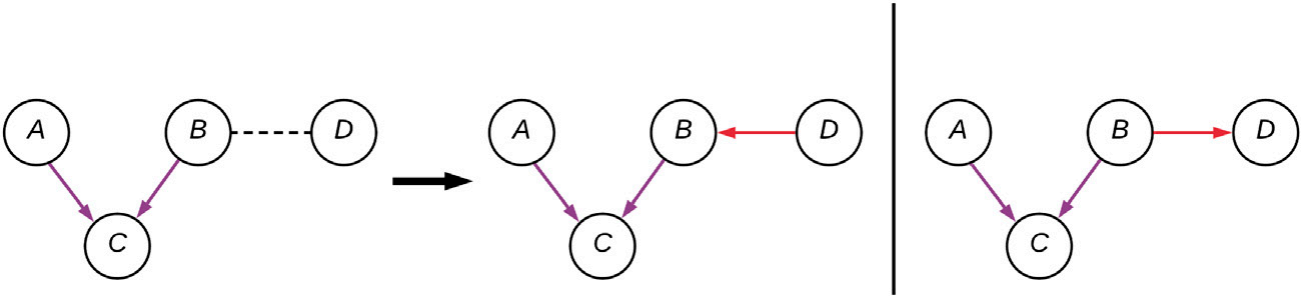
A CPDAG with four variables **(A–D)**, with two possible DAG instantiations. The edges forming the V-structure **(A–C)** are purple, and the two alternative **(B–D)** connections are in red.

A factorization can represent multiple DAGs, this set of DAGs is known as the equivalence class and are said to be Markov equivalent. BNs of the same equivalence class share the same skeleton: the underlying undirected graph, and V-structures. The skeleton of a DAG is the undirected graph resulted by ignoring every edge’s directionality. A V-structure is an unshielded common effect; that is, for the pattern of edges A → C ← B, A and B are independent (Figure 2). In this example, by having two edges pointing at it, C is said to have an in-degree of 2; A and B are the parent nodes, and C is the child node. The combination of both skeleton and V-structures is known as a complete partially directed acyclic graph, or CPDAG, and represents the equivalence class of DAGs for a factorization. Thus, we believe that how well structural learning algorithms recover the ground truth from observational data should include both skeleton and V-structure recovery.

In order for a Bayesian network to be considered causal, the parents of each of the nodes must be its direct causes. A node *A* is considered a direct cause of *C* if varying the value of *A*, while all other nodes remain unchanged, effects the distribution of *C* i.e.,:

$$P\left(C|do\left(A=a_{1}\right),do\left(B=b_{1}\right)\right)\neq P\left(C|do\left(A=a_{2}\right),do\left(B=b_{1}\right)\right)$$

Where $P\left(C|do\left(A=a_{1}\right)\right)$ is the interventional distribution; the distribution of *C* given an intervention on *A* that sets it to $a_{1}$. Additionally, the causal Markov assumption must be made to treat a BN as causal; it is assumed all common causes are present within *G*, with each node being independent conditioned on its direct causes (Hausman and Woodward, 1999).

### 2.2 Related Work

When learning a Bayesian network we are attempting to model the underlying generative model behind a given dataset. Performance of causal discovery algorithms is a function of both the distance to the underlying causal structure, as well as the distance to the true parameters. However, measuring the distance is generally not possible as the ground truth is unavailable. One strategy to obtain some expected level of Bayesian network performance, in the absence of any ground truth to compare against, is to construct a proxy of the ground-truth. Conceptually, this is similar to the previously mentioned intersection-validation method. In this method a proxy agreement graph is constructed by taking the intersection of the output from many structure learning algorithms (Viinikka et al., 2018). These algorithms are then ranked by how many samples it takes to reach this agreement graph. This forms a dependence between the selection of algorithms and the proxy, and by extension the ranking. Forming a proxy independent of algorithm choice is desirable.

Synthetic data is system-generated data which is not obtained by any direct measurement. Generally, the goal of this generated data is to mimic some real-world data, given some user-defined parameters. One can create synthetic data by two means; by modification or generation. Data can be modified, normally through anonymization, to create a synthetic dataset. Alternatively, generative models such as Generative Adversarial Networks, Variational Auto-encoders or Normalizing Flows can be sampled from to create the data (Kingma and Welling, 2013; Goodfellow et al., 2014; Rezende and Mohamed, 2015). In this study, we required a generative model which could be explicitly represented as a BN, in order to ascertain how well BN learning procedures performed. As BNs are generative models themselves, our goal is to directly create Bayesian networks with similar properties to the underlying generative model behind the real-world processes.

Previous studies have used synthetic Bayesian networks to evaluate performance of structure learning algorithms (Tasaki et al., 2015; Andrews et al., 2018; Gadetsky et al., 2020; Zhang et al., 2020; Gogoshin et al., 2020) (Table 1). These are often limited in terms of user-controllable parameters, with structures being sampled uniformly from the space of DAGs, or limited in terms of variation in topology. Other studies use standard benchmark datasets (Scutari et al., 2019; Ramanan and Natarajan, 2020). A flexible synthetic generation system would allow the user to specify many parameters which influence the BN generation, in order to match a given real dataset as closely as possible.

**TABLE 1:** Existing synthetic Bayesian network generation methods, with network attributes each method implements control over. Properties compared are those known both to vary, and to influence structure learning performance (✓) signifies a feature is implemented (✗) it is not, and (**-**) a partial implementation. Existing methods do not support latent confounding or offer control over parameter generation, and offer only random DAGs and at most one pre-defined structure type. Our work presents a step toward fully flexible structure generation, with these features the main remaining known limitations.

Method/Property	Flexible DAG Generation	Flexible Parameter Generation	Controllable levels	Latent confounding
CDG-T (ours)	**-**	**-**	✓	✗
Gogoshin et al. (2020)	**-**	✗	✓	✗
Zhang et al. (2020)	**-**	✗	✗	✗
Andrews et al. (2018)	✗	✗	✓	✗
Tasaki et al. (2015)	**-**	N/A	N/A	✗

### 2.3 Causal Datasheet Generation Tool

There are two primary goals of the Causal Datasheet. The first goal is to provide some expectation of performance given the basic, observable, characteristics of a dataset. The second goal is to provide guidance as to how many samples will be required in order to meet desired performance levels. The proof-of-concept approach we employ is described in the subsequent Section, followed by an outline of the assumptions made using this method.

#### 2.3.1 Approach

**FIGURE 3:**
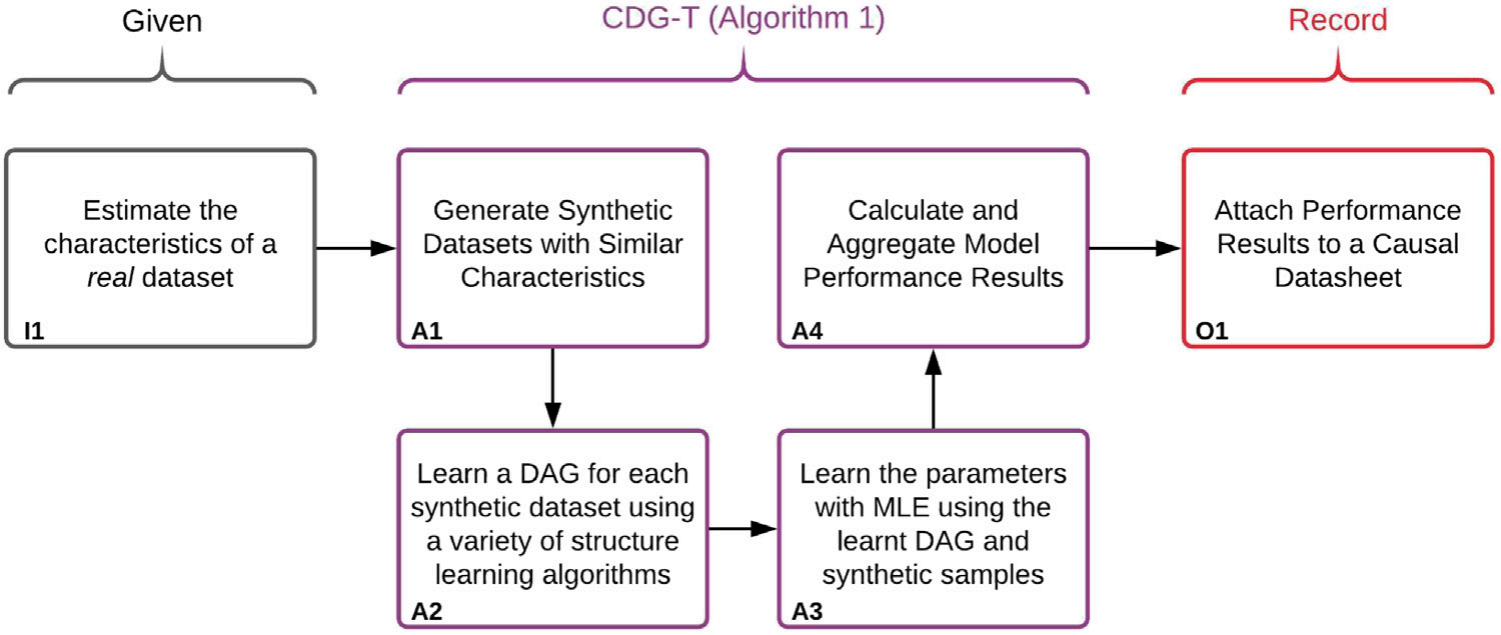
Illustration of the approach used to create the Causal Datasheets presented in Section 3. Algorithm 1 refers to the algorithm labelled *Algorithm 1* *:CDG-T Overview* below.

Our general approach is illustrated in Figure 3. In order to provide a performance estimate of structure and parameter learning for a given real dataset, we generate a set of synthetic Bayesian networks to act as a proxy for real data. Because we will have access to the ground-truths of these synthetic networks, we can calculate the performance of the structure learning, parameter learning, and any downstream estimates. Performance estimates will only be accurate so long as the generated synthetic datasets are similar enough to the given real dataset. We therefore generate synthetic BNs, and corresponding datasets, with matching observable characteristics of the real dataset. These characteristics include number of samples, variables, and levels. This corresponds to box I1 of Figure 3. In addition to the observable characteristics, there are a number of unobservable characteristics which are varied throughout the BN generation–these are discussed in Section 2.4. A small Python library which can generate and sample synthetic BNs was developed, and can be found at: https://pypi.org/project/BayNet/. An example Jupyter notebook of how to generate a datasheet can be found at: https://github.com/predictive-analytics-lab/datasheet_generation.The BN and data generation, as well as the learning and evaluation process is described in Algorithm 1, corresponding to boxes A1–A4 in Figure 3. Box A1 concerns the generation of the synthetic data, details of which can be found in Section 2.4. Box A2 and A3 concern learning a BN using the synthetic data, details of the structure learning algorithms used can be found in Section 2.5. Box A4 concerns the evaluation of the learned models, the metrics used can be found in Section 2.6.

The synthetic BN generation is performed *T* times per set of data characteristics, where *T* is a user-defined number of trials, in order to capture performance variation. While we attempt to capture as much of the space of possible BNs as we can, the number of experiments that can be performed are limited by finite computation resources. In our experiments, we set *T* to 10 to balance the total computation time for generating the data sets and learning the model spanning across configurations and to capture result uncertainty simply due to random seeding.

**Algorithm** 1: CDG-T Overview.

Given:

- The observable properties of a real dataset (sample size *n*, number of variables *p*, average levels *l*)
- The unobservable estimates of a real dataset (structure type *t*, CPT imbalance α)
- The number of trials *T*
- Hyper-parameters of structure learning algorithms

For i=1→T

1. Generate a DAG *G* with variables *V* where |V|=p according to user-specified structure-types
2. Sample number of levels for each variable from U(2,M) where l=(2+M)/2
3. Populate the parameters of the BN B=(G,Θ) with multinomial distributions $\theta_{V_{i}}$ drawn from a Dirichlet distribution with $\alpha_{V_{i}}=\frac{\alpha }{q_{V_{i}}\cdot r_{V_{i}}}$ for each variable $V_{i}\in V$
4. Draw *s* synthetic samples from synthetically created BN *B* to create synthetic dataset $X_{S}$
5. Learn a DAG using a set of structure learning algorithms using the synthetic samples
6. Learn the parameters with maximum likelihood estimation (MLE) using the learned DAG and synthetic samples
7. Record the structural performance (skeleton/v-structure precision, recall) and interventional performance (PCOR)

We envision that this extensive evaluation is synthesized into a digestible *Causal Datasheet for Existing Datasets* or *Causal Datasheet for Data Collection* format (Figure 3, Box O1). Domain experts can then assess whether this level of performance is sufficient for a particular application. Due to the flexibility of this system, we can not only construct proxies of existing datasets, but of datasets we plan to collect. In this manner, data collection can be designed around desired performance of our models. This concept is extensible to other systems with the capability to produce and evaluate synthetic data sets and structural learning algorithms. As the capability and flexibility of these systems increase, so too will the accuracy of the estimates within the Causal Datasheet.

#### 2.3.2 Assumptions

A number of assumptions are made in the generation of this synthetic data: A) we introduce no latent confounders. In order for a BN to be considered *causal*, one must assume there are no confounders absent. There are potentially complex repercussions of having confounders latent in a BN, but this is currently not examined. B) parameters are generated from a Dirichlet distribution assuming the *α* vector is uniform. The implications of this simplification are, given sufficient samples, the mean of the distributions drawn will be uniform. Therefore, generally, the marginal distributions of all nodes in the synthetic BNs will be uniform–this can make the BN learning process easier, potentially inflating performance estimates for cases where variables are highly imbalanced. This is further discussed in Section 4. Initial work has been performed to go beyond this simplification, and can be found in the supplementary material. C) it is assumed that the unobervable characteristics of a real dataset have been appropriately selected. We have assumed the used structure types can sufficiently represent the underlying DAG of a given real dataset. In the case these are incorrectly set, this could lead to incorrect performance estimates. D) we assume that synthetic data can sufficiently mimic a real dataset. Initial work has been performed to guide whether Assumptions C and D hold, and can be found in the supplementary material. These assumptions do not invalidate the concept of the Causal Datasheet, but must be kept in mind when interpreting results of a datasheet generated using CDG-T.

### 2.4 Dataset Characteristics

To study the variability of structural learning performance with different synthetic data properties, we defined two classes of dataset characteristics that can be varied to produce a distribution of synthetic data: observable and non-observable (Table 2).

**TABLE 2:** A table on the observability of the properties of BNs, as well as the values the synthetic generation tool can use.

Characteristic	Observable	Possible values
Number of Samples	✓	1–1,000,000
Number of variables	✓	1–500
Average variable levels	✓	1–10
Structure type	✗	Forest fire, IC-DAG, barabasi-albert, waxman, Small world
Maximum in-degree	✗	1–∞
α (imbalance)	✗	0–∞

Observable characteristics are those which the designer of the dataset has control and can be easily calculated (e.g., sample size and number of variables). Non-observable characteristics are properties of the underlying truth (e.g., degree distribution, type of structure, or imbalance). Non-observable characteristics can be estimated, but doing so introduces modeling assumptions. When evaluating a real-world dataset in practice, one could look up a Causal Datasheet with corresponding observable characteristics, to estimate performance uncertainty from the unobservable characteristics. Number of samples, number of variables, and average variable levels are straightforward; we describe the other characteristics below.

#### 2.4.1 Structure Type

**FIGURE 4:**
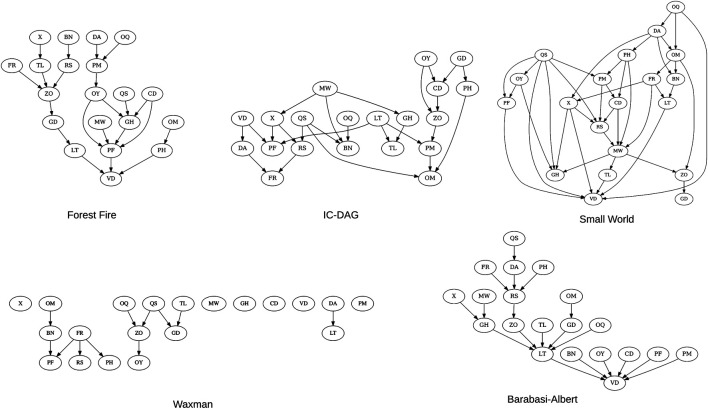
20-node examples of the five structure types used to generate synthetic BNs. As the waxman structure type is a random geometric graph, the connectivity is proportional to the number of nodes–at 20 nodes the DAG remains sparse.

We make use of five existing graph generation algorithms when creating synthetic Bayesian networks (Figure 4):

- **Forest Fire:** A growing network model which resembles how forest fires spread to nearby nodes (Leskovec et al., 2005).
- **IC-DAG:** A graph generation algorithm which samples uniformly from the set of DAGs (Ide and Cozman, 2002).
- **Barabasi-Albert** An evolving graph generation algorithm which adds edges to a new node dependent on current in-degree (Barabási and Albert, 1999).
- **Waxman:** Nodes are placed uniformly in a rectangular domain (Waxman, 1988).
- **Small-World:** A type of graph where most nodes are not direct neighbors, but the shortest path between any two nodes is generally low (Watts and Strogatz, 1998).

BNs decompose into a set of local distributions $P\left(X_{i}|{\text{Π}}_{X_{i}}\right)$. This property is utilized in structure learning algorithms; local scores or conditional independence tests are used to test a parent → child relationship. The difficulty in correctly identifying an edge is a function of the data-to-parameter ratio. The DAG has a direct effect on the number of parameters, as the higher the in-degree for a node, the more parameters it will have: $\left(r_{i}-1\right)q_{i}$, where $q_{i}$ is the product of number of parent levels. It follows then, that the DAG influences the difficulty of learning a BN by its distribution of node in-degrees. Having some control over this distribution is essential in order to complete a comprehensive evaluation. Gogoshin et al. (2020), Andrews et al. (2018), and Zhang et al. (2020) generate random networks with caps on maximum in-degree (Table 1). Tasaki et al. (2015) use graph generation algorithms in order to create synthetic BNs, but limit their use to a single type of topology. Here, we make use of multiple graph generation algorithms, allowing us to model many different *realistic* distributions of in-degrees, without having to specify them explicitly. Knowledge of what type of graphs are present in a particular domain can be incorporated by stratifying the structure type. New structure types can also be added in the case where current structure types do not sufficiency represent the topology of a specific domain.

#### 2.4.2 Maximum In-degree

Maximum in-degree is the parameter which controls the cap on the number of parents each node can have within a network. We have found structures with high in-degrees have a major effect on the performance of structure learning algorithms. Having a parameter which can control this is crucial given prior knowledge about maximum in-degree is available. Unlike other studies, in the absence of domain knowledge we did not specifically cap the maximum in-degree in addition to what structural type would implicitly generate as previously mentioned.

#### 2.4.3 Conditional Probability Table Imbalance: *α*

The CPT for each node $X_{i}$ in the synthetic Bayesian network are populated from parameters drawn from a Dirichlet distribution, with $\alpha_{i}=\frac{\alpha }{q_{i}r_{i}}$. Where $r_{i}$ is the cardinality of variable $X_{i}$, and $q_{i}$ is the product of cardinalities of parent set of $X_{i}$. A hyper-parameter, α, controls the over-all conditional imbalance, and thus connection strength, in the network. Consider an example of populating a CPT for a node with three levels and one parent. In the case the parent has two levels, two multinomial distributions must be drawn. One for each parent configuration. For example, using an *α* value of 12, applying the normalization: $\alpha_{i}=\frac{12}{3\cdot 2}=2$. Given this low value, the Dirichlet will likely draw a two low entropy multinomial distributions such as [.8,.1,.1] and [.2,.7,.1]. As these distributions are substantially different from one another, the relationship between the parent and child should be relatively easy to observe once data has been drawn conditioned on the parent value. Note that $\alpha_{i}$ is a vector $\alpha_{i}\in {\mathbb{R}}_{+}$, and the simplifying assumption has been made that it is uniform. Thus, the *α* controls the imbalance of the distributions drawn, but does not enforce any consistency as to which values are imbalanced across the conditional distribution. While we currently present results from a uniform *α* estimate within the datasheets, we have completed preliminary work to estimate a non-uniform *α* from any given dataset. Descriptions of this method, as well as results, can be found in the Supplementary Material.

### 2.5 Structure Learning Algorithms

The causal discovery step of training a causal Bayesian network is performed by structure learning algorithms (Glymour et al., 2019). In the current iteration of the Causal Datasheet three state-of-the-art structure learning algorithms are used. Each of the algorithms represents an example of constraint-based, score-based, and hybrid class of structural learning algorithms:

- **Peter-Clark (PC):** A **Constraint-based** algorithm. This algorithm starts with the graph fully connected, then uses (conditional) independence tests to iteratively prune edges. The chi-square test with mutual information is used (Spirtes et al., 2000).
- **Greedy Equivalence Search (GES):** A greedy **Score-based** algorithm, which goes through phases of adding then removing edges where doing so increases the score, alternating until the score no longer improves (Chickering, 1995). A commonly used information theoretic score, the Bayesian Information Criterion (BIC), is used (Schwarz, 1978).
- **OrderMCMC:** A **Hybrid** algorithm. This optimizes a score in the space of topological node orderings, rather than DAGs. This is an implementation of Markov Chain Monte Carlo method in the topological node ordering space (Friedman and Koller, 2003), based on modifications proposed by Kuipers et al. (2018). Each order is scored by summing the maximum score for each node, out of parent sets falling in the intersection of those permitted by the order and those in a candidate set. This set is typically initialized using a constraint-based method, such as PC, however we instead use pairwise mutual information in an effort to decouple the algorithm’s performance from that of *P*C. This candidate parent set is greedily expanded to improve recall. This combination of constrained global search with greedy means performance should be lower bounded by GES, but at much higher computation cost. For consistency with the GES algorithm the BIC score is used. We were also interested in a novel application of OrderMCMC using a recently developed score–the Quotient Normalized Maximum Likelihood (qNML) score; we included this to demonstrate differences due to choice of score (Silander et al., 2018).

These algorithms were selected in order to include one of each constraint-based, score-based, and hybrid structure learning algorithms. PC and GES were used as the constraint-based and score-based representatives as they are easily available algorithms, in terms of implementation (Scutari, 2009; Kalainathan and Goudet, 2019). OrderMCMC was used as the hybrid representative as it is the algorithm which is currently employed for the real-world examples in Section 3. The OrderMCMC implementation is currently proprietary; access can be granted from the authors on a per-request basis.

#### 2.5.1 Score Functions

Score-based and hybrid structure learning algorithms’ performance is highly dependent on choice of score function. While equivalent in the infinite data limit, the qNML and BIC scores differ significantly for small sample sizes (Silander et al., 2018). This is due to the difference in penalization; while both are based on the minimum description length (MDL) principle, they take differing approaches. The BIC takes a Bayesian approach to the penalization, using the number of parameters scaled by the log of the sample size (Schwarz, 1978); while the qNML is based on the NML (Normalized Maximum Likelihood), an exact formulation of the minimax code length regret (Grünwald and Grunwald, 2007). Both are score equivalent and free of tuning hyper-parameters.

Another score function prominent in literature is Bayesian Dirichlet equivalent uniform (BDeu) (Buntine, 1991), however it has been shown to be highly sensitive to its hyper-parameter, the effective sample size (Silander et al., 2012)–it is therefore impossible to give a reasonable estimate of performance, thus making it unsuitable for use in a datasheet.

### 2.6 Metrics

#### 2.6.1 Structural Performance

Discovery of the entire causal topology through structure learning algorithms is an appealing feature of BNs in global health settings. This sets it apart from simply testing causality between a candidate cause and the outcome interest (bivariate causal discovery), where a practitioner would be ignorant of the interaction of the system as a whole. To empirically evaluate structure learning methods with different synthetic characteristics, we measure the precision and recall of the learned structure with respect to the ground truth structure. This allowed us to separate errors into learning false edges v not identifying true edges, as opposed to quantifying aggregated structural distance measures (e.g., Structural Hamming Distance (de Jongh and Druzdzel, 2009)). For the same reason, we did not include a summarization of precision and recall, such as the F1 score. Having a clear separation of precision and recall is important in decision making; situations may arise where practitioners must favor one over the other, and the two are often a trade-off.

Structure learning algorithms estimate a DAG up to the equivalence class (CPDAG). Therefore, we do not calculate the precision and recall with respect to the true DAG, but the learned skeleton and V-structures to their ground truth counterparts. Evaluating with respect to the DAG, while helpful from an ease-of-interpretability standpoint, introduces randomly directed edges correlated to the infrequency of V-structures. This correlation can lead to misleading hypothesis when performing experiments across many different types of structure with varying prevalence of V-structures.

Precision and recall for the skeleton and V-structures of a structure are calculated in the standard manner:

$$Precision=\frac{TP}{TP+FP},\;Recall=\frac{TP}{TP+FN}$$

In the case of the skeleton true positives (TPs) are the number of undirected edges which are in both the true and learnt structure. False positives (FPs) are the number of undirected arcs which are in the learnt, yet not present in the true structure. False negatives (FNs) are the number of undirected arcs which are in the true, but not in the learnt structure. For V-structures, true positives are the number of V-structures which are present in both the learnt CPDAG and the true DAG. False positives are the number of V-structures in the learnt CPDAG while not in the true DAG. False negatives are present in the true DAG, but not the learnt CPDAG.

#### 2.6.2 Interventional Performance

One of the key uses of a causal Bayesian Network model is that, for a given outcome variable of interest, one can test hypothetical interventions on each variable. One can then compute the *interventional* odds ratio (OR) of how the outcome may change based on the intervention.

$$OR=\frac{P\left(A=a_{1}|do\left(B=b_{1}\right)\right)}{P\left(A=a_{1}|do\left(B=b_{2}\right)\right)}/\frac{P\left(A=a_{2}|do\left(B=b_{1}\right)\right)}{P\left(A=a_{2}|do\left(B=b_{2}\right)\right)}=\frac{a}{c}/\frac{b}{d}$$

The results of this intervention encompasses both the causal structure learned, and the parameters (estimated by Maximum Likelihood) of the conditional probability tables at each variable. We calculate the standard error for the odds ratios by:

$$SE=\frac{1}{aN}+\frac{1}{bN}+\frac{1}{cN}+\frac{1}{dN}$$

where *N* is the number of samples in the training data. 95% Confidence intervals are then obtained by $\text{log}\left(OR\right)\pm \sqrt{1.96}\cdot SE$.

In our Causal Datasheet we also wish to estimate how well we can approximate the true impact of interventions. A metric has been developed to measure the *proportion of correct interventional odds ratios* (PCOR) to quantify the impact of different learned structures on the interventional odds ratios. The measure was designed to provide an answer the question to practitioners: how trustworthy should these interventional odds ratios be with my dataset?

**FIGURE 5:**
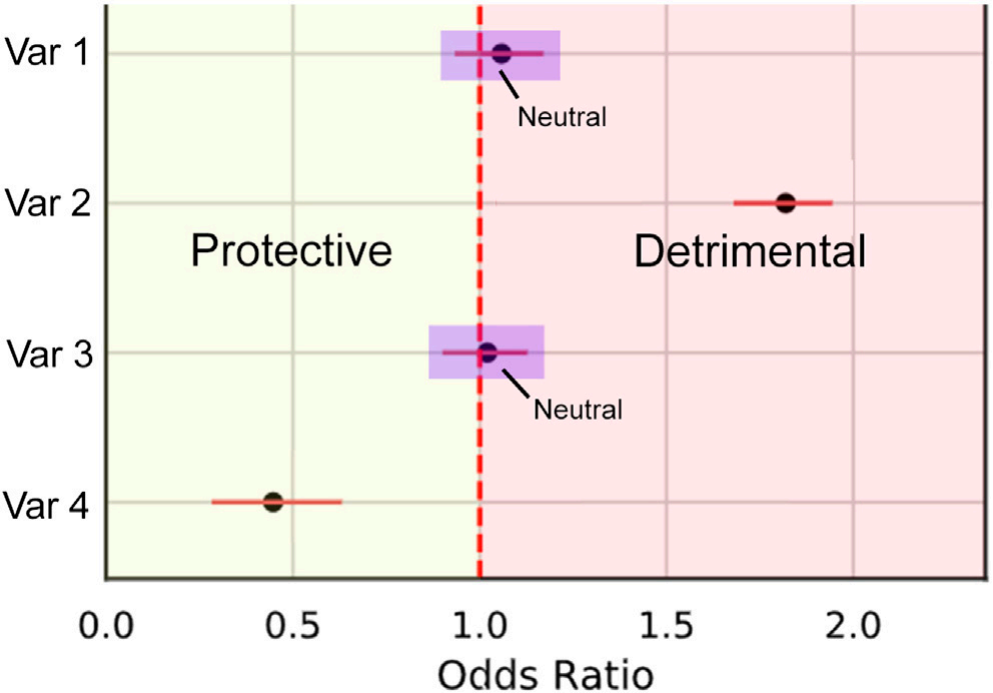
Illustration demonstrating the three categories of odds ratio *types*. Protective effects are within the bounds of the yellow Section, Detrimental within red, and neutral where the confidence intervals intersect 1 (examples within the purple boxes).

We calculate this metric by first splitting odds ratios into three types of effect: Protective (less than 1), detrimental (greater than 1), and neutral where the confidence interval crosses 1 (Figure 5). This is represented by the piecewise function in Eq. 2. The piecewise function is then used within PCOR (Eq. 1) to calculate the proportion of correctly categorized ORs.

$$\text{PCOR}\left(O,\hat{O}\right)=\frac{{\sum^{\text{​}}}_{i=1}^{\left|O\right|}\text{max}\left(f\left(O_{i}\right)\cdot f\left({\hat{O}}_{i}\right),0\right)}{{\sum^{\text{​}}}_{i=1}^{\left|O\right|}f{\left(O_{i}\right)}^{2}}$$ (1)

$$\text{with},\;f\left(O\right)=\{\begin{array}{ll} -1 & \text{if protective},\\ 0 & \text{if neutral},\\ 1 & \text{if detrimental}, \end{array}$$ (2)

where *O* is the set of odds ratios obtained by performing all possible interventions on target *T* on the true BN *B* and $\hat{O}$ are the corresponding odds ratio estimates from the learnt BN $\hat{B}$. For a synthetic Bayesian network, the target is heuristically selected as the variable with the maximum number of ancestors.

Due to variation in importance of interventions vs. outcomes, we allow the user to specify a threshold of PCOR. Recommendations from the Causal Datasheet should then be based off whether this threshold was met. Ultimately, because PCOR relies on both the structure and parameters of the network being learnt sufficiently well, these do not need to be individually assessed.

### 2.7 Causal Datasheet for Datasets

There are two types of Causal Datasheets one may follow dependent on usage: a **Causal Datasheet for Data Collection** and a **Causal Datasheet for Existing Datasets**. CDG-T is designed to allow the adjustment of numerous characteristics of synthetic data to mimic that a real-world dataset. For existing datasets, as well as the user-defined characteristics, the structure types are varied in order to capture variation in performance due to differing causal structures. For data collection, the sample size and number of variables are also varied so a user could decide what combinations of sample size and number of variables (and if applicable, structural learning algorithm) best meet the user’s analytic needs in a lookup table.

#### 2.7.1 Causal Datasheet for Data Collection

If researchers are designing their own survey or evaluation instrument, determining the sample size is a critical step. Researchers want to include a sufficient number of samples so that they can have confidence in their model results, but do not want to waste time, money, or effort by collecting unnecessarily large numbers of samples. Researchers are often constrained by budgetary concerns in low-resource settings, and adding an extra thousand samples may end up costing thousands of extra dollars in effort.

In traditional public health and medical studies, a priori power analysis is the preferred tool for quantifying the samples size needed to sufficiently detect changes, treatment effects, or associations between variables (Pourhoseingholi et al., 2013). The Datasheet for Data Collection can fill a similar role for BN analysis. In the Datasheet for Data Collection, users can specify a range of desired, potential sample size and variable size and then estimate performance for models of interest.

The resulting datasheet is organized in four main sections: Recommendations, Proportion of Correct Odds Ratios, Skeleton Precision and Recall, and V-structure Precision and Recall. The *recommendations* Section outlines the main takeaways and suggestions from the data creator/curator who examined the expected performance across a given range of sample sizes and variable sizes, and should be treated as a guide. *Proportion of Correct Odds Ratios*, *Skeleton* and *V-structure performance* sections allow a user to look up combinations of sample sizes and variable sizes that would fit the analysis requirement for correctness of intervention effect, correct edges ignoring directions, and correct edges considering directions accordingly. The resulting datasheet produces surface plots so a user can explore in either or both sample size or variable size dimensions that may maximize expected (median) model learning performance given the user’s desired study design. One should also consider the variation of measure performances - lower variation is better, as it suggests an algorithm is less sensitive to the unobserved characteristics. This is provided as Inter-Quartile Range (IQR) in tables corresponding to the surface plots. If two algorithms, and/or two combination of sample size and variable sizes result in similar distribution of performance measures (median and IQR), then one may choose either one. This datasheet provides a general guidance for determining the best combination of sample size and number of variables to maximize BN model performance.

#### 2.7.2 Causal Datasheet for Existing Datasets

The Datasheet for Existing Datasets assists researchers in determining the suitability of using BNs to meet research objectives, given they already know their sample size and number of variables. The goal of this datasheet is to provide insight into how much confidence they should have in BN models learnt from this dataset. Additionally, researchers may use this datasheet to determine which algorithms to use, and how much of a ground truth DAG they can expect to recover. For example, researchers in global health often rely on previously deployed datasets to generate insights. A public health researcher might want to use data from an existing survey to generate causal insights around health decisions in a particular area. They could use the Datasheet for Existing Datasets to evaluate its suitability for generating insights and to inform feature engineering decisions.

This type of Causal Datasheet starts with the data characteristics used to generate the synthetic data sets and to compute the various metrics followed with recommendations to a potential audience who may be considering using the data set to infer causal relationships. This leading section outlines the main takeaways and suggestions from the data creator/curator who examined the data set. The main body of the datasheet is then broken down to 1. Correctness of causal effects, 2. Learning the Skeleton, 3. Learning the Direction, 4. Improving with More Samples, and 5. Improving with Less Variables. The goal is to offer the potential data user the best guesses of expected performance from different perspectives, recognizing that different applications may call for choosing an algorithm or feature engineering approach optimized for different measures. These measures are described above and on a scale from 0 to 1, with 1 being perfect. They are depicted with violin plots to aid assessment of expected uncertainties given a measure. If there are multiple “bumps” in the violin plots, in our experience, this is because of different structural learning performance as results of learning different data sets generated with different structure types. If desired, performance stratification by structure type and further investigation may be warranted; however this is beyond the scope of the current work. In *Correctness of Causal Effects*, the PCOR metrics is used as an attempt to estimate how well we can approximate intervention impact using different structural learning algorithms where 1 represents all protective, neutral and detrimental intervention effects are likely to be correctly captured. In *Learning the Skeleton* Section, the precision and recall of edges, ignoring the directions, are presented. In *Learning the Direction* Section, edge directions (as a V-structures) are also considered in the precision and recall. Skeleton learning performance measures are usually better than V-structure learning performance; if one is not too concerned with learning the causal directions, one may be satisfied with good skeleton learning performance alone. Moreover, one may care more about recall over precision (e.g., in an exploratory study aiming to identify all potential relationships between variables) or vice versa. Lastly, the Improving with More Samples and Improving with Less Variables sections show how much more data or how many variables to reduce to improve structure learning performance for different algorithms, assuming relevant causal statistics is not degraded by the reduction of variables in the latter.

### 2.8 Meta-Feature Similarity Between Synthetic Datasets and Existing Dataset of Interest

Our approach makes the assumption that we could tune the characteristics of the synthetic datasets such that the synthetic datasets are similar to the dataset of interest, and thus it is reasonable to suggest that expected performance on the dataset of interest could be approximated by metrics computed on the synthetic datasets. How similar are the synthetic datasets to the existing dataset of interest? We should not compare them using only data characteristics that are used to generate the synthetic datasets. Instead, we borrow the concept of defining meta-features and computing dataset similarities from the Bayesian optimization hyperparameter initialization literature (Wistuba et al., 2015). For categorical datasets, information theoretic meta-features (i.e., Shannon’s entropy and Concentration Coefficient) are computed for both the real and synthetic datasets (Michie et al., 1994; Alexandros and Melanie, 2001). The similarity between them then is computed as the mean cosine similarity, with 0 being completely dissimilar and 1 being identical. For additional details, please refer to the Supplementary Material.

## 3 Results

### 3.1 Causal Datasheet for Data Collection Example: Survey Design of a Study of Sexual and Reproductive Health

We first used a Datasheet for Data Collection, generated using CDG-T, to determine the appropriate sample size of a survey we deployed in Madhya Pradesh, India (Supplementary Material A). In 2019, we had the opportunity to use the CDG-T to determine the sample size of a large-scale survey of sexual and reproductive health (SRH) we conducted in Madhya Pradesh, India. Determining sample size of this study was important because it had implications for the overall budget and timeline of our project. Typically we wish to have a survey to capture as many variable as possible (provided the survey is not too long) with as few samples as possible. Our survey sought to quantify a wide range of causal drivers around family planning decisions. These variables included demographics, knowledge and beliefs, risk perceptions, past experiences, and structural determinants such as accessibility. We estimated that we would have between 30–60 variables that would be critical causal drivers of sexual and reproductive health decisions. From previous work, we estimated that causal variables would have, on average, three levels. We decided to use the Datasheet for Data Collection to determine model performance for between 5,000 and 15,000 survey respondents before commissioning the field study. While we had determined that 5,000 respondents was likely a large enough sample to have sufficient power for predictive regression models, we did not know whether this sample size would have sufficient performance for a causal Bayesian network model. The range of 5,000 to 15,000 samples represented the budget constraints of our survey.

For simplicity, we varied the potential number of variables to be included in the model and the potential sample sizes while keep other synthetic data property constant (Table 3).

**TABLE 3:** The values used for each property when creating synthetic BNs (and their associated datasets) for the SRH datasheet. Combinatorial total of 175 property values, Each configuration of properties was repeated 10 times. In total 1750 Bayesian networks and datasets were created.

Property	Values
Variables	30, 35, 40, 45, 50, 55, 60
Samples	5,000, 7,500, 10,000, 12,500, 15,000
Average levels	3
Structure types	Forest fire, barabasi-albert, IC-DAG, waxman, Small-world
Maximum in-degree	Uncapped
α	20

The datasheet revealed insights around the optimal sample size for our study. We found that, in general, the OrderMCMC algorithm was the best for PCOR, skeleton precision and recall, and V-structure precision (Table 4) and recall (Table 5). When comparing model performance metrics, we found that 5,000 samples would likely not be enough to build robust BN models for designing interventions because, across all numbers of variables, the V-structure recall was low (<0.42, GES and PC) or was high but had high IQR with both OrderMCMC instantiations > 0.40. Our datasheet showed that as we increased sample size, the IQR of V-structure recall for the OrderMCMC algorithm decreased. In order to have better confidence in our Bayesian network models, we determined that we would need a sample of around 15,000 respondents to balance our desire of having at least 50 variables while minimizing the IQR of V-structure recall (Table 5).

**TABLE 4:** Pivot Table of V-structure precision. Rows stratify by number of variables. Columns are over samples size. V-structure Precision performance is provided as: *Median (IQR)*. Highest precision in each sample/variable combination is in bold.

Number of variables	Algorithm	5,000	7,500	10,000	12,500	15,000
30	GES	0.37 (0.31)	0.39 (0.31)	0.47 (0.37)	0.39 (0.31)	0.41 (0.31)
30	OrderMCMC (BIC)	**1.00** (**0.04)**	**1.00** (**0.00)**	1.00 (0.04)	1.00 (0.02)	**1.00** (**0.00)**
30	OrderMCMC (qNML)	1.00 (0.65)	**1.00** (**0.00)**	**1.00** (**0.00)**	**1.00** (**0.00)**	**1.00** (**0.00)**
30	PC	0.86 (0.24)	0.89 (0.13)	0.89 (0.13)	0.89 (0.12)	0.89 (0.13)
40	GES	0.37 (0.24)	0.36 (0.29)	0.36 (0.21)	0.38 (0.27)	0.32 (0.37)
40	OrderMCMC (BIC)	**1.00** (**0.03)**	**1.00** (**0.02)**	1.00 (0.02)	1.00 (0.03)	1.00 (0.02)
40	OrderMCMC (qNML)	0.97 (0.50)	1.00 (0.02)	1.00 (0.00)	1.00 (0.00)	1.00 (0.00)
40	PC	0.89 (0.16)	0.87 (0.13)	0.88 (0.15)	0.89 (0.14)	0.88 (0.16)
50	GES	0.32 (0.20)	0.33 (0.32)	0.35 (0.25)	0.32 (0.28)	0.34 (0.30)
50	OrderMCMC (BIC)	0.98 (0.04)	1.00 (0.03)	1.00 (0.03)	0.99 (0.04)	1.00 (0.03)
50	OrderMCMC (qNML)	0.97 (0.61)	0.99 (0.06)	1.00 (0.01)	1.00 (0.01)	1.00 (0.00)
50	PC	0.86 (0.16)	0.87 (0.12)	0.88 (0.12)	0.88 (0.16)	0.88 (0.15)
60	GES	0.34 (0.20)	0.34 (0.22)	0.32 (0.24)	0.30 (0.26)	0.34 (0.25)
60	OrderMCMC (BIC)	**0.98** (**0.03)**	**0.99** (**0.03)**	**1.00** (**0.02)**	0.98 (0.03)	0.99 (0.03)
60	OrderMCMC (qNML)	0.93 (0.50)	0.98 (0.23)	**1.00** (**0.02)**	**1.00** (**0.02)**	**1.00** (**0.02)**
60	PC	0.86 (0.20)	0.87 (0.20)	0.86 (0.17)	0.90 (0.17)	0.88 (0.16)

**TABLE 5:** Pivot Table of V-structure recall. Rows stratify by number of variables. Columns are over samples size. V-structure Recall performance is provided as: *Median (IQR)*. Highest recall in each sample/variable combination is in bold.

Number of variables	Algorithm	5,000	7,500	10,000	12,500	15,000
30	GES	0.21 (0.25)	0.28 (0.33)	0.30 (0.34)	0.31 (0.25)	0.33 (0.36)
30	OrderMCMC (BIC)	0.94 (0.33)	0.95 (0.28)	0.95 (0.24)	0.99 (0.21)	0.99 (0.17)
30	OrderMCMC (qNML)	**1.00** (**0.02)**	**1.00** (**0.00)**	**1.00** (**0.00)**	**1.00** (**0.00)**	**1.00** (**0.00)**
30	PC	0.43 (0.49)	0.49 (0.49)	0.54 (0.42)	0.56 (0.44)	0.56 (0.45)
40	GES	0.26 (0.19)	0.31 (0.25)	0.30 (0.26)	0.33 (0.25)	0.36 (0.23)
40	OrderMCMC (BIC)	0.84 (0.47)	0.91 (0.43)	0.92 (0.43)	0.94 (0.39)	0.97 (0.28)
40	OrderMCMC (qNML)	**1.00** (**0.20)**	**1.00** (**0.03)**	**1.00** (**0.03)**	**1.00** (**0.00)**	**1.00** (**0.00)**
40	PC	0.37 (0.26)	0.41 (0.33)	0.47 (0.23)	0.46 (0.28)	0.48 (0.27)
50	GES	0.25 (0.17)	0.28 (0.13)	0.32 (0.20)	0.32 (0.17)	0.36 (0.22)
50	OrderMCMC (BIC)	0.86 (0.42)	0.90 (0.43)	0.91 (0.40)	0.93 (0.39)	0.94 (0.38)
50	OrderMCMC (qNML)	**0.97** (**0.40)**	**1.00** (**0.33)**	**1.00** (**0.19)**	**1.00** (**0.21)**	**1.00** (**0.05)**
50	PC	0.36 (0.17)	0.38 (0.22)	0.39 (0.31)	0.40 (0.32)	0.39 (0.30)
60	GES	0.24 (0.17)	0.27 (0.18)	0.29 (0.17)	0.30 (0.21)	0.33 (0.26)
60	OrderMCMC (BIC)	0.72 (0.39)	0.80 (0.38)	0.84 (0.35)	0.84 (0.34)	0.86 (0.35)
60	OrderMCMC (qNML)	**0.93** (**0.34)**	**0.98** (**0.33)**	**0.99** (**0.31)**	**0.98** (**0.29)**	**1.00** (**0.30)**
60	PC	0.34 (0.29)	0.35 (0.33)	0.35 (0.34)	0.38 (0.35)	0.39 (0.34)

Fortunately, our budget constraints allowed us to expand our sample size to meet this constraint. However, in many cases, organizations operating in LMICs would not be able to treble their sample size. Here, the CDG-T also provides useful advice. For example, if our sample size remained at 5,000, reducing the number of causal variables from 60 to 30 would cause V-structure recall to increase for all algorithms and V-structure IQR to decrease. This would significantly improve confidence in the produced BN models, but with the implication that a potentially different analytical question may be necessary.

The CDG-T Datasheet for Data Collection provides useful information even if researchers decide that they do not want to reduce variables or increase sample size by estimating the performance of a DAG before a survey is carried out. This allows researchers to know what kind of insights and results they will be able to generate.

### 3.2 Causal Datasheet for Existing Datasets Example: Analysis of an Existing Global Health Survey (Surgo Household Dataset)

As the second example, we generated a Causal Datasheet for a global development dataset we administered in Uttar Pradesh, India in 2016 (Smittenaar et al., 2020) (Supplementary Material B). For simplicity, we refer to this dataset as Surgo Household survey or SHH. It sought to quantify household reproductive, maternal, neonatal, and child health (RMNCH) journeys and to understand the drivers of various RMNCH behaviors. In all, we surveyed over 5,000 women on various RMNCH behaviors and outcomes. From this survey, we initially identified 41 variables we thought represented critical causal drivers of RMNCH outcomes and behaviors such as birth delivery locations and early breastfeeding initiation. We were interested in understanding which interventions might be most important for different health outcomes. While it was possible to use our datasets to generate DAGs, we could not validate their structures, nor could we assign confidence to graphs generated using different structural learning algorithms.

Using survey dataset characteristics, we generated synthetic dataset experiments with similar properties (Table 6). Using a method described in Section 2.8, we computed information theoretic similarity between the synthetic datasets and the SHH data; the result is that they are indeed similar with a similarity score of 0.89. This is supportive of the assumption that the expected performance on the SHH data can be reasonably approximated by computing the metrics on the corresponding synthetic datasets.

**TABLE 6:** The values used for each property when creating synthetic BNs (and their associated datasets) for the SHH datasheet. Combinatorial total of 80 property values, Each configuration of properties was repeated 10 times. In total 800 Bayesian networks and datasets were created. Italicized values were only used when presenting results pertaining to increasing samples or decreasing variables.

Property	Values
Variables	40, *30, 20, 10*
Samples	5,000, *7,500, 10,000, 12,500*
Average levels	3
Structure types	Forest fire, barabasi-albert, IC-DAG, waxman, Small-world
Maximum in-degree	Uncapped
α	20

**FIGURE 6:**
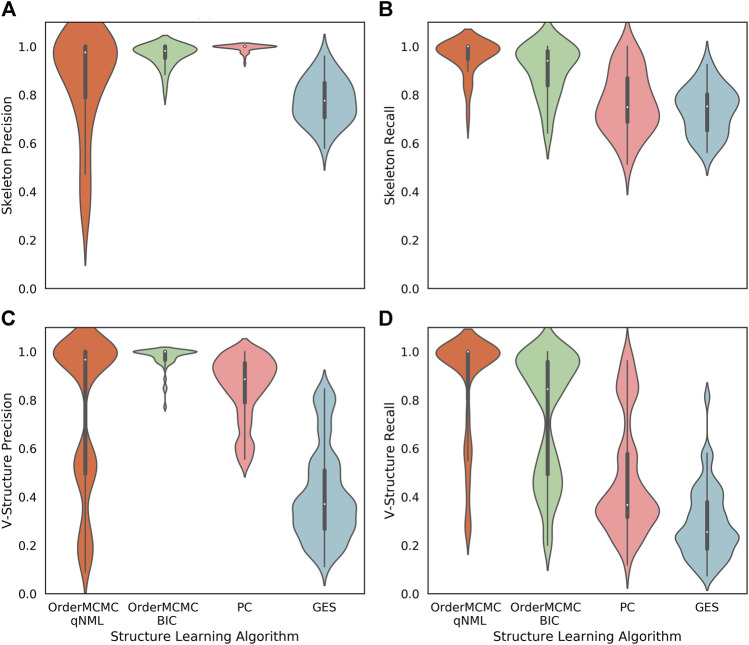
Performance obtained running the structure learning algorithms on the datasets produced for the Surgo Household datasheet (Datasheet for Existing Datasets). The width of the violin plots are the kernel density estimates of the distribution, the white dots represent the median, and the vertical thick and thin lines represent the IQRs and ranges respectively. **(A)**: Skeleton Precision; **(B)**: Skeleton Recall; **(C)**: V-structure Precision; **(D)**: V-structure Recall. From this figure we can see good skeleton performance can be obtained using OrderMCMC, but V-Structures are more likely to be correct (precision) when using the BIC and less likely to be missed (recall) when using the qNML score.

**FIGURE 7:**
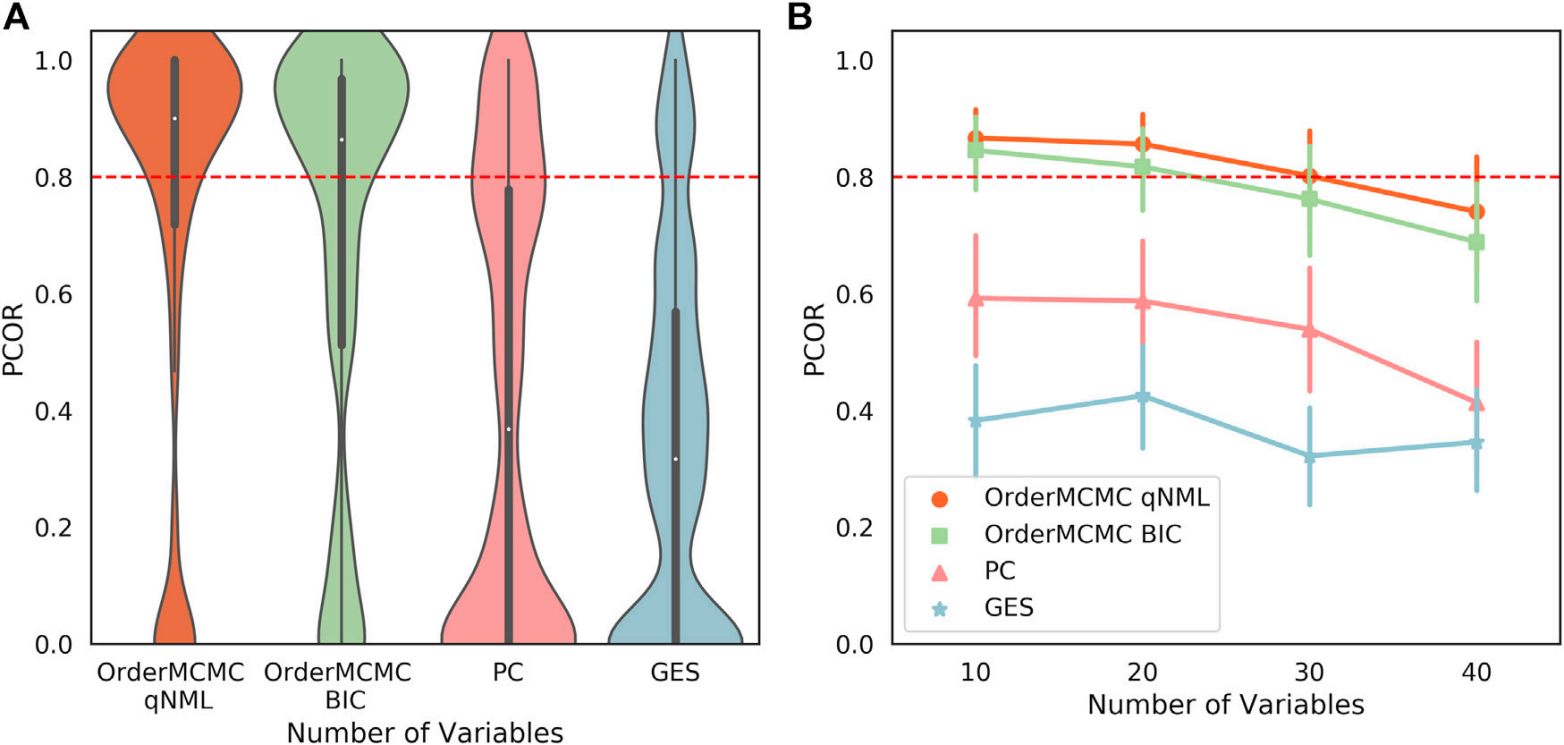
**(A)**: The PCOR score obtained running the structure learning algorithms on the datasets produced for the Surgo Household dataset datasheet. Performance for OrderMCMC is generally above the set threshold. There are cases where the there are no paths to the target variable, causing the PCOR to be 0. **(B)**: The PCOR scores obtained by reducing the number of variables. Median performance passes the threshold on the OrderMCMC results when variables are ≤ 20.

The expected BN algorithm skeleton (Figures 6A,B), V-structure (Figures 6C,D), and PCOR score (Figure 7A) were then attached to the datasheet for each of the structure learning algorithms. As our primary goal was to successfully simulate interventions, we set a threshold of 0.8 on the PCOR score. Meeting this threshold would imply we could have reasonable confidence in our model and the estimates it produced.

The outputs from the Causal Datasheet provided a number of key insights for this dataset:

1. While all structure learning algorithms could achieve high skeleton precision and recall (Figures 6A,B), the OrderMCMC algorithms (with either BIC or qNML) had superior median predicted performance for V-structure precision and recall (Figures 6C,D).
2. The median PCOR for OrderMCMC (qNML) (0.89) and OrderMCMC (BIC) met our threshold test of 0.8 (Figure 7A). There are cases when the PCOR is 0 due to target variables being determined as independent during structure learning. This is not a major concern as it will be clear to a practitioner when this has occurred once the DAG has been visualized.
3. Decreasing the number of variables from 40 to 20 could improve the mean V-structure recall by ≈0.08 (Supplementary Material B) and PCOR by (Figure 7B).
4. Structure type, specifically the distribution of in-degree, has a large effect on expected performance levels, particularly on V-structure precision (Figure 6C). This leads to a multi-modal distribution of performance where similar structures are grouped. If a practitioner can ascertain the ground truth structure type or the distribution of in-degree, even if he/she cannot ascertain the ground truth structure itself, the uncertainty of the performance estimation can be reduced.

Specifically on ground truth skeleton recovery, we found that PC may provide marginally higher performance on skeleton precision (1 vs. 0.98), but performs poorly in recall by comparison with OrderMCMC (qNML) (0.75 vs. 1). OrderMCMC (BIC) had the highest median skeleton precision at 1. OrderMCMC (qNML), PC and GES were at 0.97, 0.91 and 0.36 respectively. However, the precision with PC is less sensitive with a IQR of 0.02, whereas OrderMCMC (qNML) was 0.21 and OrderMCMC (BIC) was 0.05.

On ground truth V-structure recovery, OrderMCMC (BIC) performs best in terms of V-structure precision, but suffers with V-structure recall compared to OrderMCMC (qNML). Particularly on structure types with higher in-degrees, forest fire and Barabasi-Albert. PC recall for V-structures is much worse than OrderMCMC (qNML) (0.37 vs. 1). Overall GES with BIC is a poor performer for our needs, especially when V-structure is concerned despite having the same score function as OrderMCMC (BIC).

These insights were invaluable for decision making in the relevant context and showed us that we would need to further reduce our number of variables or seek expert input before we could have confidence in our understanding of the effects of interventions on maternal health outcomes. The results also suggested that the OrderMCMC algorithm qNML would generally provide the best overall model performance among those tested. Ultimately, given we had a clear outcome variable of interest, we used multivariate regression to select 18 out of 40 variables based on significance of regression coefficients from the original dataset; this reduction in the number of variables allowed us to have more confidence in our resulting DAG structures. It should be pointed out that there are many general-purpose feature selection schemes, but feature selection with the intent for subsequent causal structural discovery is not well understood and beyond the scope of this study (Guyon et al., 2007).

### 3.3 Causal Datasheet for Existing Datasets Example: ALARM

As a third example, a Causal Datasheet for the well-known ALARM dataset was generated for the purpose of validating the estimates being produced by CDG-T (Supplement C). The characteristics of this dataset were approximated, mimicking how a researcher might use the Causal Dataset Generation Tool. The ALARM dataset has 37 variables, and an average of 2.8 levels per variable (Beinlich et al., 1989). Aside from a few binary variables, most variables have ordinal values. In this test case a sample size of 5,000 was used. Synthetic BNs with similar characteristics were then generated, the exact values used can be found in Table 7. Using a method described in Section 2.8, we computed information theoretic similarity between the synthetic datasets and the ALARM data; the result is that they are indeed similar with a similarity score of 0.91. This is supportive of the assumption that the expected performance on the ALARM dataset can be reasonably approximated by computing the metrics on the corresponding synthetic datasets. Experiments using these Synthetic BNs were then performed, with the results summarized in the datasheet.

**TABLE 7:** The values used for each property when creating synthetic BNs (and their associated datasets) for the ALARM datasheet. Combinatorial total of five property values, Each configuration of properties was repeated 10 times. In total 50 Bayesian networks and datasets were created.

Property	Values
Variables	40
Samples	5,000
Average levels	3
Structure types	Forest fire, barabasi-albert, IC-DAG, waxman, Small-world
Maximum in-degree	Uncapped
α	6

While the synethetic datasets are similar to the ALARM dataset, they are not identical. As the ALARM dataset has a known corresponding ground-truth, it can be used to test the limitations of our current approach due the assumptions we make when generating synethetic datasets.

One such assumption is that when sampling parameters from a Dirichlet distribution we have assumed *α* is uniform, this means (on average) the marginal distributions of the variables will be balanced. Imbalanced marginal distributions can degrade structure learning performance, as information supporting conditional dependence becomes more scarce with the same amount of data.

**FIGURE 8:**
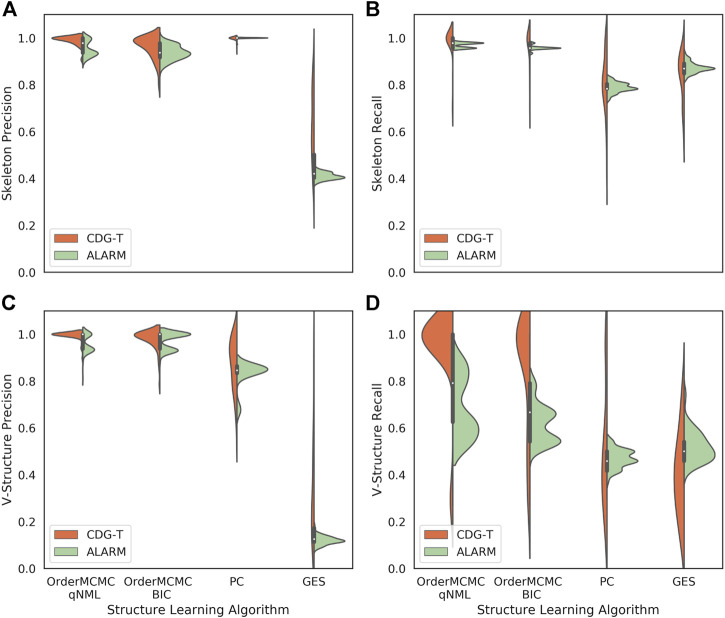
The performance obtained on CDG-T datasets vs. the ALARM dataset over 100 runs. **(A)**: Skeleton Precision; **(B)**: Skeleton Recall; **(C)**: V-structure Precision; **(D)**: V-structure Recall. There is a general alignment on the skeleton estimates, but V-Structure Recall is overestimated for the OrderMCMC methods.

Comparing the CDG-T estimate generated with the uniform *α* assumption to the actual performance obtained on the ALARM dataset shows general alignment with the PC and GES algorithms, but an overestimation of performance with OrderMCMC (Figure 8). This difference can be observed when looking at V-structure recall (Figure 8D). Our preliminary analysis suggests that when *α* is not assumed to be uniform, such misalignment decreases. Moreover, information theoretic similarity also increases (Supplementary Material D) from 0.91 to 0.99.

## 4 Discussion

Having a Causal Datasheet that describes the expected performance in recovering ground truth structures for any given dataset can be tremendously valuable to both machine learning scientists and practitioners. We were particularly interested in scenarios where data characteristics are sub-optimal for data-driven causal BN learning, which is often the case for LMIC scenarios. This perspective differs from other evaluative reviews of algorithm in the sense that we are not only concerned with different structural learning algorithms’ (and score function choices’) maximum capacity to recover the ground truth, but also how they differ in more constrained cases (Raghu et al., 2018). We have shown how Causal Datasheets can aid in the planning of studies that have the analytical goal of causal discovery and inference, and in analysis of studies after existing data have been collected. Our general approach of creating synthetic datasets that approximate the real-world data should accommodate other causal inference methods such as Neyman-Rubin causal models (the potential outcomes framework) in theory (Rubin, 2005).

In addition to the number of variables and sample size demonstrated in the case studies, we have also observed that extreme imbalance of levels, in-degree and structure type all affect structural learning performance. In practice, even observable characteristics may be beyond the modeler’s control. Sometimes the sample size is restricted by resources such as survey budget or similar data have already been collected. Sometimes a variable may be very imbalanced (e.g., very few unhealthy samples vs. many healthy samples). Often, data are collected with specific questions in mind and may not contain all the right variables for another specific outcome of interest. However, upon referring to such Causal Datasheets, there may be scenarios where seemingly imperfect dataset could still yield useful insights, given a tolerable level of error. Moreover, one’s tolerance may be different for precision and recall errors. In constrained scenarios, our results suggest that practitioners may be able to increase algorithm performances by additional feature engineering or transformation of the data by reducing the number of variables for example. However, one should be cautioned against too much data processing as it runs the risk of transforming the ground truth represented by the data as well.

We briefly discuss the potential impact of our Causal Datasheet work on algorithmic fairness research. Gebru et al. (2018) advocated that every dataset is accompanied with a datasheet to promote transparency and accountability, including to highlight if the dataset has unwanted bias toward a particular demographic. It is important for us to understand how and why demographic information, especially protected characteristics (e.g., race, gender, age), influences other variables in a dataset. Causal reasoning has recently been shown to be a powerful tool for understanding sources of algorithmic bias, and for mitigating bias in an algorithmic decision system (Chiappa and Isaac, 2018; Loftus et al., 2018; Sharmanska et al., 2020). Most existing causality-based algorithmic fairness methods require knowledge of the causal graph. One option is to learn causal structure from observational data. It is important to acknowledge that potentially very misleading conclusions might be drawn if incorrect causal structure is used (Kilbertus et al., 2020). Our Causal Datasheet can be used to help researchers and practitioners assess whether they can have confidence in the inferred structure.

### 4.1 Assumptions and Limitations

While we believe the datasheet has utility in its current form, there are still a number of improvements to be made. Assumptions are made when building the Causal Datasheets. In order to present the results from synthetic experiments as performance which can be expected on empirical data, we must assume that synthetic data can act as a proxy for empirical data. Furthermore, the synthetic data in use can be improved upon; there may be other pertinent data characteristics that we had not considered or considered but incorrectly assumed. These include the assumed ground truth structure types, where the five graph generation algorithms used offer no guarantee of orthogonality. While using known structure types can be an advantage if a practitioner suspects their DAG may be of a particular distribution, using multiple can clearly bias results if they generate in-degree distributions which are too similar. A future direction of research would be to use generative graph models which when seeded with an initial DAG can preserve degree distribution, among other properties (Leskovec and Faloutsos, 2007). How to form a DAG as a seed in an unbiased and useful way is non-trivial. A high-recall DAG could be used in an attempt to provide an upper-bound on difficulty, under the assumption the in-degree distribution would be at least on par with reality. Alternatively an agreement graph, as in the Intersection-Validation paper, could be used as a seed to provide a DAG with less bias to any one structure learning algorithm. For simplicity we have also assumed that the conditional imbalance parameter applies to the entire dataset, but it is entirely possible that a real dataset has a large variance around the imbalance of parameters. Validating our tool with ALARM demonstrates there are special cases which are not yet entirely modeled in our synthetic data generation. The current simplifying assumption of uniform *α* values when sampling parameters from a Dirichlet distribution can clearly lead to overestimation of performance in some scenarios. Development of *α*-estimation techniques, or other methods of incorporating non-uniform *α* values is a clear next step. Some initial work can be found in the supplementary material. Introducing further modeling assumptions, whether by generative graph or *α* estimation techniques, can increase the specificity of the provided estimates. However, introducing bias in this way must be done with caution. As it could yield certain, yet incorrect, performance estimates. A method of determining whether introduced assumptions are correct, and to address the gap between real and synthetic data must be developed. Some initial work on this can again be found in the supplementary material. Others have shown that algorithm outputs are sensitive to hyper-parameters specific to that algorithm. For example, BDeu is a popular score but it is highly sensitive to its only hyper-parameter, the equivalent sample size (Scutari, 2018). This is part of the reason we included qNML and BIC in the current study as they do not have hyper-parameters. Estimation of hyper-parameters is often not trivial and may challenging to generalize across a spectrum of real-world data. Additionally, we have only considered BN here, which cannot accommodate cyclical causal relationships. Finally, we have assumed that the input datasets had no latent confounders and the datasets are at least meet the interventional sufficiency criteria (Pearl, 2009; Peters et al., 2017), which is known to be a problem.

There are also practical limitations as well. We had considered data with discrete variables only; however this approach can be extended to algorithms that deal with continuous variables as well. We did not consider computational power needed for different algorithms. While we bear a faithful optimism that computation power of current hardware will increase to eventually overcome this barrier, this is a useful addition to the Causal Datasheet. Similarly the computation time to generate the synthetic data sets is also highly dependent on the hardware. However, since it took about 1 min to generate 50 synthetic data sets (40 variables, 5,000 samples, five structure types and 10 repetitions) on a workstation equipped with an AMD EPYC 7742 CPU and 256 GB of RAM, we think this will not be a big problem for most in the long run as cloud computing solutions become democratized and cheaper. With ten repeats (i.e., *T* = 10), our largest set of experiments had 1,750 datasets to generate, learn, and evaluate; taking around 40 h to complete. Running times are highly dependent on configuration, as well as the machine being used, and should be selected appropriately for individual circumstances. Whether 10 repeats of each experiment are required, or is sufficient, remains unknown. While the synthetic BN and datasets generation is inexpensive (time-wise), structure learning on the data is not, and by-far takes the most time of any component in our datasheet generation pipeline. For example, while data generation for the ALARM dataset takes 1 min, the structure learning takes close to an hour. Another clear direction of future work is to perform analysis to determine when enough experiments have taken place to reach some performance convergence. We have made assessments based on purely precision and recall of the ground truth, ignoring the fact that our OrderMCMC implementation takes longer than PC and GES; however there may be circumstances where computational speed outweighs the benefit of accuracy gains. Real-world data often come with missingness that are either random (MAR), completely at random (MCAR), and missing not at random (MNAR) (Rubin, 1976). We have developed the capacity to produce synthetic datasets with missingness. Determining missingness characteristics along with the appropriate imputation method for use in the datasheet is a future research direction. Lastly, we were inspired by the problem of inferring causality from global development datasets and have estimated the range of data characteristics subjectively in that domain. By all means, the range of data characteristics considered in this study may be very different for a different sub-domain. For example, the number of variables for agricultural data may be many more than that of a disease treatment survey. We leave these theoretical and practical limitations as potential areas for improvement to further the usage of Bayesian networks in practice.

In summary, a standard practice of reporting projected range of causal discovery and inference performance can help practitioners 1) during the experimental design phase, when they are interested in designing experiments with characteristics suitable for BN analysis, 2) during the analysis phase, when they are interested in choosing optimal structural learning algorithms and assigning confidence to DAGs, and 3) at the policy level, when they must justify their insights generated from BN analysis. We believe that this type of evaluation should be a vital component to a general causal discovery and inference work flow.

## Data Availability

The original contributions presented in the study are included in the article/Supplementary Material, further inquiries can be directed to the corresponding author.
