# Supplementary material for: Causal Datasheet for Datasets: An Evaluation Guide for Real-World Data Analysis and Data Collection Design Using Bayesian Networks
Source: Front Artif Intell. 2021 Apr 14;4:612551. doi: 10.3389/frai.2021.612551 (PMC8320747; doi:10.3389/frai.2021.612551)
Supplement: Supplementary file 1 [file datasheet1.pdf]

# Supplementary Material

## INTRODUCTION

The supplementary material is composed of five items:

A) Data Collection example: survey design of a study of sexual and reproductive health – corresponding to section 3.1 in the main text
B) Existing Datasets example: Analysis of an existing global health survey – corresponding to section 3.2
C) Existing Datasets example: ALARM – corresponding to section 3.3
D) Non-uniform $\alpha$ Estimation and Meta-Feature Similarity
E) Additional Uniform $\alpha$ Estimates

Items A, B, and C correspond to the datasheets discussed in the results section of the main text. How these should be interpreted can be found in section 2.7. Item D is the work we have performed to address the limitations discussed in section 4 of the main text, along with some initial results. E provides extra results not given in the main text.

# (Supplementary Material A) Causal Datasheet for Data Collection: Sample size 5,000 - 15,000, Number of Variables 30 - 60

**Recommendations**

The following are a list of recommendations for using Dataset to learn a Bayesian network:

- PCOR SUMMARY
- Skeleton precision and recall - minimum combination to reach some threshold? Or something similar
- V-structure performance

## Proportion of Correct Odds Ratios

The **Proportion of Correct Odds Ratios**(PCOR) measures the proportion of interventional odds ratios a learnt BN correctly estimates. This metric is calculated by first splitting odds ratios into three types of effects: protective (less than 1), detrimental (greater than 1), and neutral (uncertainty crosses 1). A matrix of these three effects is constructed for both the learned and true odds ratios, as show below.

| Learnt/True | Protective | Neutral | Detrimental |
|---|---|---|---|
| Protective | 1 | 0 | 0 |
| Neutral | 0 | 0 | 0 |
| Detrimental | 0 | 0 | 1 |

The above matrix is the one used in this evaluation. If the true effect is protective or detrimental, it is counted as *correct* when the learned odds ratio matches. Matching a neutral effect is not considered as a *correct* odds ratio, as we have found neutral effects can artificially inflate the results and create a false correlation.

The table below show the median PCOR of different algorithms for each combination of specified parameters.In general, users should select combinations of Algorithm, Number of Variables, and Sample Size that maximize PCOR performance and minimize IQR.

| Number of Variables | Algorithm | 5000 | 7500 | 10000 | 12500 | 15000 |
|---|---|---|---|---|---|---|
| 30 | GES | 0.27 (0.54) | 0.36 (0.51) | 0.35 (0.53) | 0.34 (0.44) | 0.4 (0.45) |
| 30 | OrderMCMC (BIC) | 0.91 (0.26) | 0.93 (0.23) | 0.94 (0.22) | 0.96 (0.17) | 0.92 (0.29) |
| 30 | OrderMCMC (qNML) | 0.92 (0.18) | 0.95 (0.2) | 0.94 (0.14) | 0.94 (0.14) | 0.96 (0.16) |
| 30 | PC | 0.54 (0.74) | 0.7 (0.63) | 0.69 (0.6) | 0.75 (0.56) | 0.68 (0.65) |
| 40 | GES | 0.32 (0.57) | 0.33 (0.55) | 0.35 (0.45) | 0.3 (0.45) | 0.42 (0.5) |
| 40 | OrderMCMC (BIC) | 0.86 (0.46) | 0.88 (0.29) | 0.91 (0.33) | 0.9 (0.28) | 0.94 (0.32) |
| 40 | OrderMCMC (qNML) | 0.9 (0.28) | 0.9 (0.2) | 0.91 (0.12) | 0.93 (0.15) | 0.94 (0.18) |
| 40 | PC | 0.37 (0.78) | 0.45 (0.83) | 0.49 (0.67) | 0.48 (0.67) | 0.54 (0.68) |
| 50 | GES | 0.33 (0.53) | 0.24 (0.52) | 0.35 (0.51) | 0.35 (0.42) | 0.34 (0.65) |
| 50 | OrderMCMC (BIC) | 0.8 (0.65) | 0.87 (0.35) | 0.89 (0.33) | 0.87 (0.35) | 0.92 (0.32) |
| 50 | OrderMCMC (qNML) | 0.91 (0.22) | 0.94 (0.19) | 0.92 (0.18) | 0.92 (0.2) | 0.93 (0.14) |
| 50 | PC | 0.53 (0.82) | 0.46 (0.76) | 0.42 (0.82) | 0.39 (0.73) | 0.41 (0.8) |
| 60 | GES | 0.43 (0.72) | 0.33 (0.61) | 0.34 (0.58) | 0.47 (0.53) | 0.36 (0.57) |
| 60 | OrderMCMC (BIC) | 0.88 (0.8) | 0.85 (0.74) | 0.9 (0.58) | 0.88 (0.51) | 0.89 (0.43) |
| 60 | OrderMCMC (qNML) | 0.89 (0.29) | 0.92 (0.19) | 0.96 (0.28) | 0.94 (0.39) | 0.92 (0.26) |
| 60 | PC | 0.52 (0.82) | 0.46 (0.71) | 0.43 (0.85) | 0.44 (0.72) | 0.4 (0.82) |

Table 1: Pivot Table of **PCOR** performance. Rows stratify by number of variables / algorithm used. Columns are over samples size. PCOR performance is provided as: *Median (IQR)*

The surface plot graphs expected PCOR performance for different combinations of algorithm, number of variables, and sample size. The color of the graph surface indicates the algorithm that is expected to have the highest performance for a given combination of parameters.

Below is a summary of expected PCOR performance:

- Item 1
- Insight 2

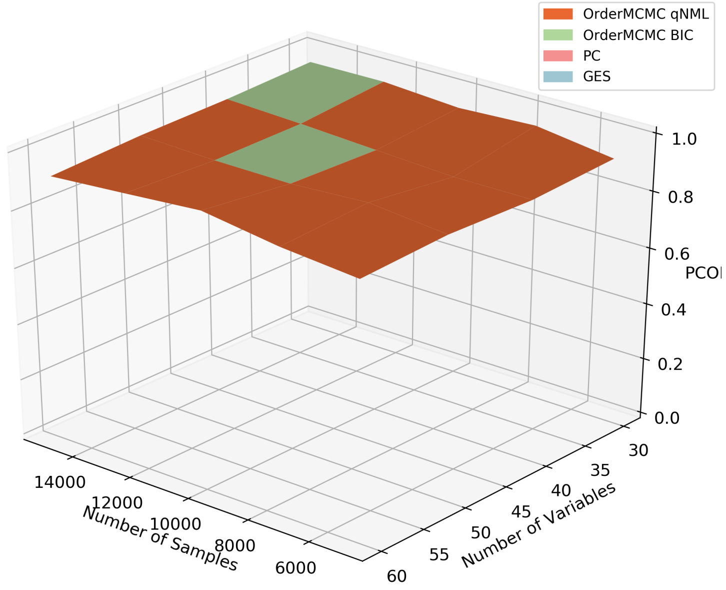


Figure 1: Surface plot of PCOR performance

# Skeleton Precision

**Precision**, also known as the positive predictive value, is defined as the number of correct edge predictions made divided by all edge predictions made. The upper bound of precision is one, which corresponds to when all predicted true cases are correct. The lower bound is zero, which indicates that none of the predicted cases were correct.

The table below show the median Skeleton Precision of different algorithms for each combination of specified parameters. In general, users should select combinations of Algorithm, Number of Variables, and Sample Size that maximize Skeleton Precision performance and minimize IQR.

| Number of Variables | Algorithm | 5000 | 7500 | 10000 | 12500 | 15000 |
|---|---|---|---|---|---|---|
| 30 | GES | 0.82 (0.15) | 0.81 (0.17) | 0.79 (0.21) | 0.78 (0.22) | 0.77 (0.21) |
| 30 | OrderMCMC (BIC) | 0.97 (0.04) | 1.0 (0.03) | 1.0 (0.03) | 1.0 (0.03) | 1.0 (0.03) |
| 30 | OrderMCMC (qNML) | 1.0 (0.29) | 1.0 (0.0) | 1.0 (0.0) | 1.0 (0.0) | 1.0 (0.0) |
| 30 | PC | 1.0 (0.0) | 1.0 (0.0) | 1.0 (0.0) | 1.0 (0.02) | 1.0 (0.0) |
| 40 | GES | 0.78 (0.14) | 0.75 (0.16) | 0.77 (0.16) | 0.73 (0.17) | 0.72 (0.18) |
| 40 | OrderMCMC (BIC) | 0.98 (0.05) | 0.98 (0.03) | 0.99 (0.03) | 0.98 (0.04) | 1.0 (0.02) |
| 40 | OrderMCMC (qNML) | 0.98 (0.21) | 1.0 (0.03) | 1.0 (0.02) | 1.0 (0.01) | 1.0 (0.0) |
| 40 | PC | 1.0 (0.0) | 1.0 (0.0) | 1.0 (0.0) | 1.0 (0.0) | 1.0 (0.0) |
| 50 | GES | 0.76 (0.15) | 0.75 (0.17) | 0.73 (0.15) | 0.72 (0.17) | 0.71 (0.2) |
| 50 | OrderMCMC (BIC) | 0.98 (0.04) | 0.98 (0.04) | 0.98 (0.04) | 0.98 (0.04) | 0.99 (0.04) |
| 50 | OrderMCMC (qNML) | 0.97 (0.15) | 0.99 (0.05) | 1.0 (0.02) | 1.0 (0.02) | 1.0 (0.01) |
| 50 | PC | 1.0 (0.0) | 1.0 (0.0) | 1.0 (0.0) | 1.0 (0.0) | 1.0 (0.0) |
| 60 | GES | 0.74 (0.11) | 0.75 (0.12) | 0.73 (0.15) | 0.71 (0.17) | 0.71 (0.2) |
| 60 | OrderMCMC (BIC) | 0.96 (0.05) | 0.97 (0.05) | 0.99 (0.03) | 0.98 (0.04) | 0.98 (0.02) |
| 60 | OrderMCMC (qNML) | 0.96 (0.25) | 0.99 (0.07) | 0.99 (0.02) | 0.99 (0.01) | 1.0 (0.02) |
| 60 | PC | 1.0 (0.01) | 1.0 (0.0) | 1.0 (0.0) | 1.0 (0.02) | 1.0 (0.01) |

Table 2: Pivot Table of **Skeleton Precision**. Rows stratify by number of variables / algorithm used. Columns are over samples size. Skeleton Precision performance is provided as: *Median (IQR)*

The surface plot graphs expected Skeleton Precision performance for different combinations of algorithm, number of variables, and sample size. The color of the graph surface indicates the algorithm that is expected to have the highest performance for a given combination of parameters.
Below is a summary of expected Skeleton Precision performance:

- At **any** of the given operating characteristics, **very high precision** can be expected
- The **PC** algorithm should be used if precision is a priority as it overall has the lowest IQR

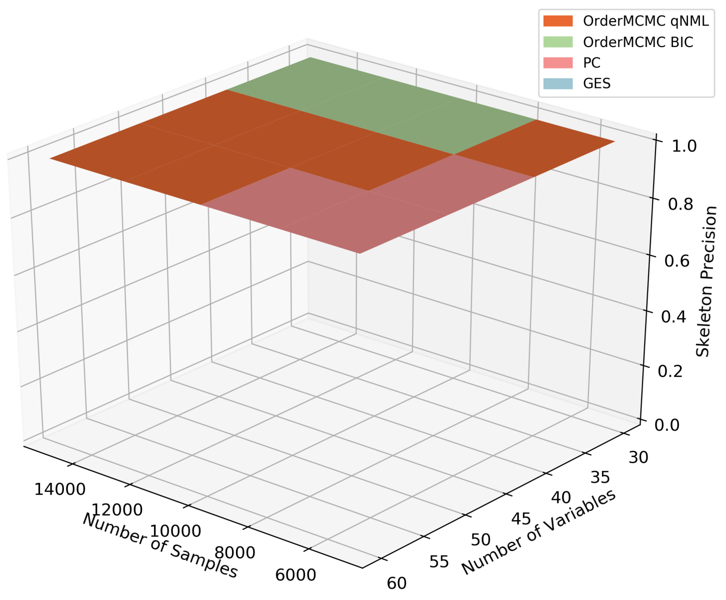


Figure 2: Surface plot of Skeleton Precision

# Skeleton Recall

**Recall** is the proportion of predicted positive cases over true positive cases. The upper bound of recall is one, corresponding to all true positive values being predicted. The lower bound is zero, indicating none of the true positive values have been captured.

The table below show the median Skeleton Recall of different algorithms for each combination of specified parameters.In general, users should select combinations of Algorithm, Number of Variables, and Sample Size that maximize Skeleton Recall performance and minimize IQR.

| Number of Variables | Algorithm | 5000 | 7500 | 10000 | 12500 | 15000 |
|---|---|---|---|---|---|---|
| 30 | GES | 0.78 (0.13) | 0.8 (0.15) | 0.82 (0.12) | 0.84 (0.14) | 0.84 (0.14) |
| 30 | OrderMCMC (BIC) | 0.95 (0.13) | 0.97 (0.11) | 0.97 (0.07) | 0.97 (0.07) | 0.98 (0.06) |
| 30 | OrderMCMC (qNML) | 1.0 (0.02) | 1.0 (0.02) | 1.0 (0.0) | 1.0 (0.0) | 1.0 (0.0) |
| 30 | PC | 0.78 (0.21) | 0.83 (0.23) | 0.84 (0.2) | 0.84 (0.21) | 0.85 (0.22) |
| 40 | GES | 0.75 (0.15) | 0.8 (0.14) | 0.79 (0.11) | 0.81 (0.14) | 0.82 (0.13) |
| 40 | OrderMCMC (BIC) | 0.94 (0.14) | 0.96 (0.11) | 0.97 (0.1) | 0.97 (0.1) | 0.98 (0.06) |
| 40 | OrderMCMC (qNML) | 1.0 (0.05) | 1.0 (0.04) | 1.0 (0.02) | 1.0 (0.02) | 1.0 (0.01) |
| 40 | PC | 0.75 (0.18) | 0.77 (0.18) | 0.78 (0.18) | 0.79 (0.16) | 0.8 (0.18) |
| 50 | GES | 0.73 (0.16) | 0.77 (0.14) | 0.79 (0.12) | 0.82 (0.12) | 0.82 (0.14) |
| 50 | OrderMCMC (BIC) | 0.93 (0.15) | 0.97 (0.14) | 0.97 (0.13) | 0.97 (0.15) | 0.97 (0.12) |
| 50 | OrderMCMC (qNML) | 0.98 (0.12) | 1.0 (0.09) | 1.0 (0.12) | 1.0 (0.07) | 1.0 (0.04) |
| 50 | PC | 0.73 (0.17) | 0.73 (0.19) | 0.75 (0.22) | 0.75 (0.23) | 0.74 (0.2) |
| 60 | GES | 0.71 (0.13) | 0.76 (0.14) | 0.76 (0.14) | 0.79 (0.11) | 0.81 (0.14) |
| 60 | OrderMCMC (BIC) | 0.9 (0.14) | 0.93 (0.13) | 0.94 (0.12) | 0.94 (0.11) | 0.95 (0.1) |
| 60 | OrderMCMC (qNML) | 0.97 (0.09) | 0.99 (0.11) | 1.0 (0.1) | 0.99 (0.1) | 1.0 (0.11) |
| 60 | PC | 0.71 (0.17) | 0.74 (0.18) | 0.73 (0.15) | 0.74 (0.19) | 0.74 (0.2) |

Table 3: Pivot Table of **Skeleton Recall**. Rows stratify by number of variables / algorithm used. Columns are over samples size. Skeleton Recall performance is provided as: *Median (IQR)*

The surface plot graphs expected Skeleton Recall performance for different combinations of algorithm, number of variables, and sample size. The color of the graph surface indicates the algorithm that is expected to have the highest performance for a given combination of parameters.

Below is a summary of expected Skeleton Recall performance:

- The **OrderMCMC (qNML)** algorithm obtains the best skeleton recall at every operating characteristic
- At 5,000 samples: recall reduces from 1 at 30 variables, to 0.971 at 60 variables.
- Increasing the number of samples from 5,000 to 15,000 improves the recall to a median of 1 at 60 variables.

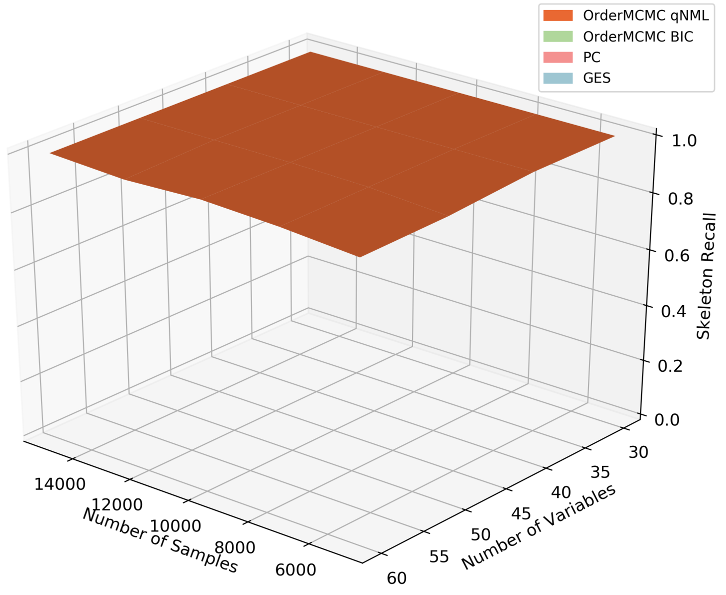


Figure 3: Surface plot of Skeleton Recall

# V-Structure Precision

The precision with respect to V-Structures is the proportion of learnt V-Structures which are present in the true CPDAG.

The table below show the median V-Structure Precision of different algorithms for each combination of specified parameters.In general, users should select combinations of Algorithm, Number of Variables, and Sample Size that maximize V-Structure Precision performance and minimize IQR.

| Number of Variables | Algorithm | 5000 | 7500 | 10000 | 12500 | 15000 |
|---|---|---|---|---|---|---|
| 30 | GES | 0.37 (0.31) | 0.39 (0.31) | 0.47 (0.37) | 0.39 (0.31) | 0.41 (0.31) |
| 30 | OrderMCMC (BIC) | 1.0 (0.04) | 1.0 (0.0) | 1.0 (0.04) | 1.0 (0.02) | 1.0 (0.0) |
| 30 | OrderMCMC (qNML) | 1.0 (0.65) | 1.0 (0.0) | 1.0 (0.0) | 1.0 (0.0) | 1.0 (0.0) |
| 30 | PC | 0.86 (0.24) | 0.89 (0.13) | 0.89 (0.13) | 0.89 (0.12) | 0.89 (0.13) |
| 40 | GES | 0.37 (0.24) | 0.36 (0.29) | 0.36 (0.21) | 0.38 (0.27) | 0.32 (0.37) |
| 40 | OrderMCMC (BIC) | 1.0 (0.03) | 1.0 (0.02) | 1.0 (0.02) | 1.0 (0.03) | 1.0 (0.02) |
| 40 | OrderMCMC (qNML) | 0.97 (0.5) | 1.0 (0.02) | 1.0 (0.0) | 1.0 (0.0) | 1.0 (0.0) |
| 40 | PC | 0.89 (0.16) | 0.87 (0.13) | 0.88 (0.15) | 0.89 (0.14) | 0.88 (0.16) |
| 50 | GES | 0.32 (0.2) | 0.33 (0.32) | 0.35 (0.25) | 0.32 (0.28) | 0.34 (0.3) |
| 50 | OrderMCMC (BIC) | 0.98 (0.04) | 1.0 (0.03) | 1.0 (0.03) | 0.99 (0.04) | 1.0 (0.03) |
| 50 | OrderMCMC (qNML) | 0.97 (0.61) | 0.99 (0.06) | 1.0 (0.01) | 1.0 (0.01) | 1.0 (0.0) |
| 50 | PC | 0.86 (0.16) | 0.87 (0.12) | 0.88 (0.12) | 0.88 (0.16) | 0.88 (0.15) |
| 60 | GES | 0.34 (0.2) | 0.34 (0.22) | 0.32 (0.24) | 0.3 (0.26) | 0.34 (0.25) |
| 60 | OrderMCMC (BIC) | 0.98 (0.03) | 0.99 (0.03) | 1.0 (0.02) | 0.98 (0.03) | 0.99 (0.03) |
| 60 | OrderMCMC (qNML) | 0.93 (0.5) | 0.98 (0.23) | 1.0 (0.02) | 1.0 (0.02) | 1.0 (0.02) |
| 60 | PC | 0.86 (0.2) | 0.87 (0.2) | 0.86 (0.17) | 0.9 (0.17) | 0.88 (0.16) |

Table 4: Pivot Table of **V-Structure Precision**. Rows stratify by number of variables / algorithm used. Columns are over samples size. V-Structure Precision performance is provided as: *Median (IQR)*

The surface plot graphs expected V-Structure Precision performance for different combinations of algorithm, number of variables, and sample size. The color of the graph surface indicates the algorithm that is expected to have the highest performance for a given combination of parameters.

Below is a summary of expected V-Structure Precision performance:

- **OrderMCMC (BIC)** should be used in order to maximize V-Structure Precision.

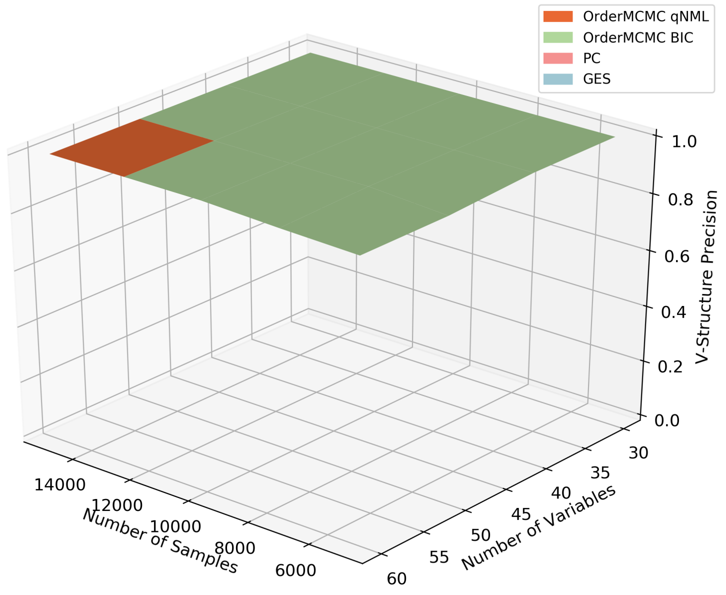


Figure 4: Surface plot of V-Structure Precision

## V-Structure Recall

Recall of V-Structures is the proportion of the true V-Structures that are present in the predicted case.

The table below show the median V-Structure Precision of different algorithms for each combination of specified parameters.In general, users should select combinations of Algorithm, Number of Variables, and Sample Size that maximize V-Structure Precision performance and minimize IQR.

| Number of Variables | Algorithm | 5000 | 7500 | 10000 | 12500 | 15000 |
|---|---|---|---|---|---|---|
| 30 | GES | 0.21 (0.25) | 0.28 (0.33) | 0.3 (0.34) | 0.31 (0.25) | 0.33 (0.36) |
| 30 | OrderMCMC (BIC) | 0.94 (0.33) | 0.95 (0.28) | 0.95 (0.24) | 0.99 (0.21) | 0.99 (0.17) |
| 30 | OrderMCMC (qNML) | 1.0 (0.02) | 1.0 (0.0) | 1.0 (0.0) | 1.0 (0.0) | 1.0 (0.0) |
| 30 | PC | 0.43 (0.49) | 0.49 (0.49) | 0.54 (0.42) | 0.56 (0.44) | 0.56 (0.45) |
| 40 | GES | 0.26 (0.19) | 0.31 (0.25) | 0.3 (0.26) | 0.33 (0.25) | 0.36 (0.23) |
| 40 | OrderMCMC (BIC) | 0.84 (0.47) | 0.91 (0.43) | 0.92 (0.43) | 0.94 (0.39) | 0.97 (0.28) |
| 40 | OrderMCMC (qNML) | 1.0 (0.2) | 1.0 (0.03) | 1.0 (0.03) | 1.0 (0.0) | 1.0 (0.0) |
| 40 | PC | 0.37 (0.26) | 0.41 (0.33) | 0.47 (0.23) | 0.46 (0.28) | 0.48 (0.27) |
| 50 | GES | 0.25 (0.17) | 0.28 (0.13) | 0.32 (0.2) | 0.32 (0.17) | 0.36 (0.22) |
| 50 | OrderMCMC (BIC) | 0.86 (0.42) | 0.9 (0.43) | 0.91 (0.4) | 0.93 (0.39) | 0.94 (0.38) |
| 50 | OrderMCMC (qNML) | 0.97 (0.4) | 1.0 (0.33) | 1.0 (0.19) | 1.0 (0.21) | 1.0 (0.05) |
| 50 | PC | 0.36 (0.17) | 0.38 (0.22) | 0.39 (0.31) | 0.4 (0.32) | 0.39 (0.3) |
| 60 | GES | 0.24 (0.17) | 0.27 (0.18) | 0.29 (0.17) | 0.3 (0.21) | 0.33 (0.26) |
| 60 | OrderMCMC (BIC) | 0.72 (0.39) | 0.8 (0.38) | 0.84 (0.35) | 0.84 (0.34) | 0.86 (0.35) |
| 60 | OrderMCMC (qNML) | 0.93 (0.34) | 0.98 (0.33) | 0.99 (0.31) | 0.98 (0.29) | 1.0 (0.3) |
| 60 | PC | 0.34 (0.29) | 0.35 (0.33) | 0.35 (0.34) | 0.38 (0.35) | 0.39 (0.34) |

Table 5: Pivot Table of **V-Structure Recall**. Rows stratify by number of variables / algorithm used. Columns are over samples size. V-Structure Recall performance is provided as: *Median (IQR)*

The surface plot graphs expected V-Structure Recall performance for different combinations of algorithm, number of variables, and sample size. The color of the graph surface indicates the algorithm that is expected to have the highest performance for a given combination of parameters.

Below is a summary of expected V-Structure Recall performance:

- **OrderMCMC (qNML)** should be used in order to maximize V-Structure recall.

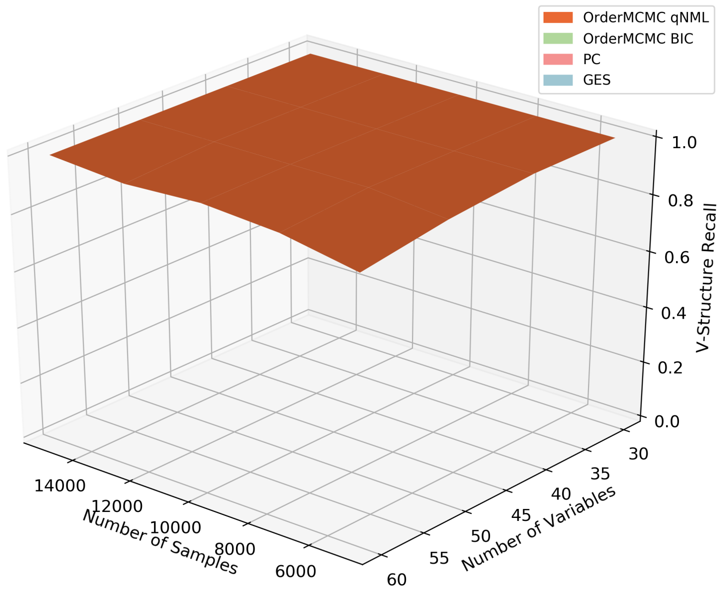


Figure 5: Surface plot of V-Structure Recall

# (Supplementary Material B) Causal Datasheet for Maternal Health in Uttar Pradesh Dataset

**Input Data Characteristics**

**Variables:** 40

**Samples:** 5,000

**Average Levels:** 3

**Alpha:** 20

**Maximum In-degree:** $\infty$

**Structure Types:** Forest Fire, IC-DAG, Barabasi-Albert, Waxman, Small World

## Recommendations

The following are a list of recommendations for using Maternal Health in Uttar Pradesh Dataset to learn a Bayesian network:

- Given the **0.80 PCOR threshold** supplied by the user, the Maternal Health in Uttar Pradesh Dataset **can** be used for learning a Bayesian network without further samples, constraints, or expert input.
- It is recommended the **OrderMCMC (qNML)** algorithm is used as this obtains the best performance.
- **PC** should be used if **skeleton precision** is the priority, while **OrderMCMC (qNML)** should be used if **skeleton recall** is the priority
- **OrderMCMC (qNML)** should be used if successfully capturing V-Structures is a priority

## 1 Correctness of Causal Effects

The **Proportion of Correct Odds Ratios**(PCOR) measures the proportion of interventional odds ratios a learnt BN correctly estimates. This metric is calculated by first splitting odds ratios into three types of effects: protective (less than 1), detrimental (greater than 1), and neutral (uncertainty crosses 1). A matrix of these three effects is constructed for both the learned and true odds ratios, as show below.

| Learnt/True | Protective | Neutral | Detrimental |
|---|---|---|---|
| Protective | 1 | 0 | 0 |
| Neutral | 0 | 0 | 0 |
| Detrimental | 0 | 0 | 1 |

The above matrix is the one used in this evaluation. If the true effect is protective or detrimental, it is counted as *correct* when the learned odds ratio matches. Matching a neutral effect is not considered as a *correct* odds ratio, as we have found neutral effects can artificially inflate the results and create a false correlation.

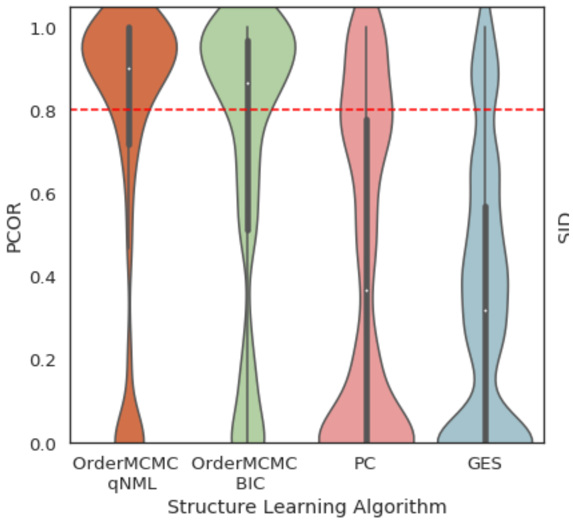


- Median PCOR using the **OrderMCMC (qNML)** algorithm = 0.9 (IQR = 0.28)
- Median PCOR using the **OrderMCMC (BIC)** algorithm = 0.86 (IQR = 0.46)
- Median PCOR using the **PC** algorithm = 0.37 (IQR = 0.78)
- Median PCOR using the **GES** algorithm = 0.32 (IQR = 0.57)

## 2 Learning the Skeleton

The following are plots showing what skeleton structure learning performance to expect given Maternal Health in Uttar Pradesh Dataset's properties:

### 2.1 Skeleton Precision

**Precision**, also known as the positive predictive value, is defined as the number of correct edge predictions made divided by all edge predictions made. The upper bound of precision is one, which corresponds to when all predicted true cases are correct. The lower bound is zero, which indicates that none of the predicted cases were correct.

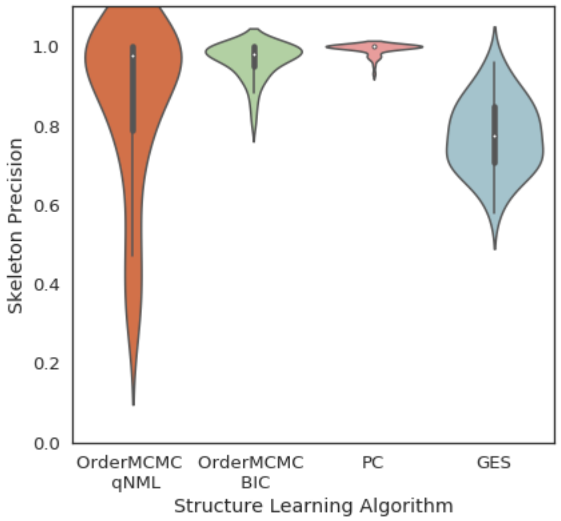


- Median skeleton precision using the **OrderMCMC (qNML)** algorithm = 0.98 (IQR = 0.21)
- Median skeleton precision using the **OrderMCMC (BIC)** algorithm = 0.98 (IQR = 0.05)
- Median skeleton precision using the **PC** algorithm = 1.0 (IQR = 0.0)
- Median skeleton precision using the **GES** algorithm = 0.78 (IQR = 0.14)

### 2.2 Skeleton Recall

**Recall** is the proportion of predicted positive cases over true positive cases. The upper bound of recall is one, corresponding to all true positive values being predicted. The lower bound is zero, indicating none of the true positive values have been captured.

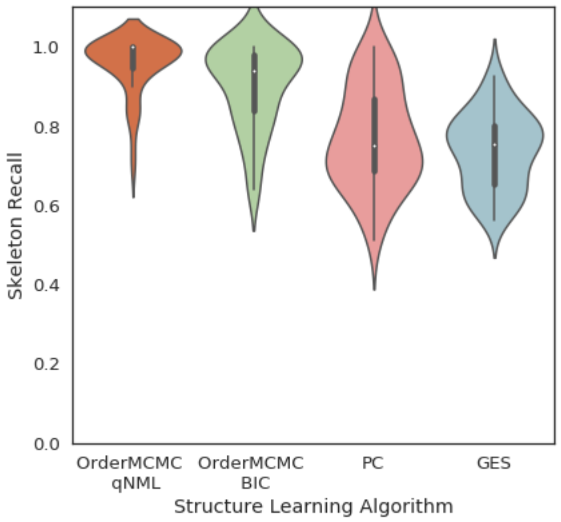


- Median skeleton recall using the **OrderMCMC (qNML)** algorithm = 1.0 (IQR = 0.05)
- Median skeleton recall using the **OrderMCMC (BIC)** algorithm = 0.94 (IQR = 0.14)
- Median skeleton recall using the **PC** algorithm = 0.75 (IQR = 0.18)
- Median skeleton recall using the **GES** algorithm = 0.75 (IQR = 0.15)

## 3 Learning the Direction

The following are plots showing what V-structure structure learning performance to expect given Maternal Health in Uttar Pradesh Dataset properties:

### 3.1 V-Structure Precision

The precision with respect to V-Structures is the proportion of learnt V-Structures which are present in the true CPDAG.

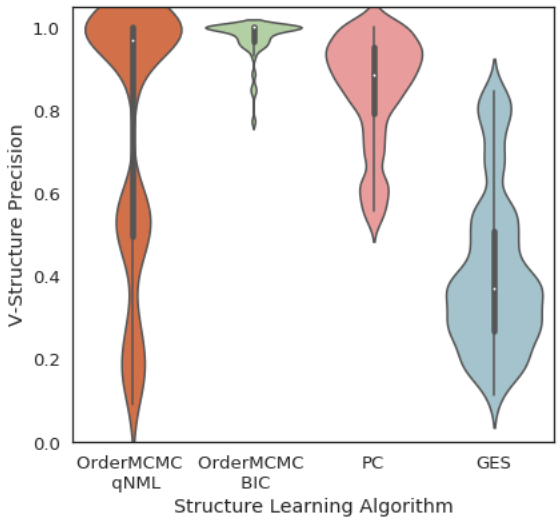


- Median V-Structure precision using the **OrderMCMC (qNML)** algorithm = 0.97 (IQR = 0.5)
- Median V-Structure precision using the **OrderMCMC (BIC)** algorithm = 1.0 (IQR = 0.03)
- Median V-Structure precision using the **PC** algorithm = 0.89 (IQR = 0.16)
- Median V-Structure precision using the **GES** algorithm = 0.37 (IQR = 0.24)

### 3.2 V-Structure Recall

Recall of V-Structures is the proportion of the true V-Structures that are present in the predicted case.

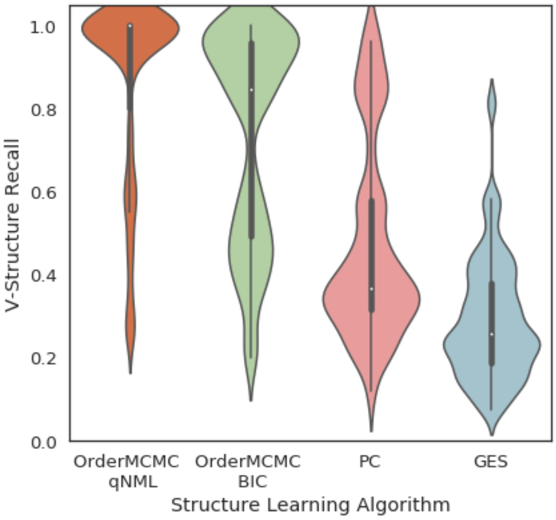


- Median V-Structure recall using the **OrderMCMC (qNML)** algorithm = 1.0 (IQR = 0.2)
- Median V-Structure recall using the **OrderMCMC (BIC)** algorithm = 0.84 (IQR = 0.47)
- Median V-Structure recall using the **PC** algorithm = 0.37 (IQR = 0.26)
- Median V-Structure recall using the **GES** algorithm = 0.26 (IQR = 0.19)

# 4 Improving with More Samples

If the performance shown is this datasheet is not acceptable, improvements can generally be made by adding more samples. The following plot shows expected performance gain with further samples for Maternal Health in Uttar Pradesh Dataset:

## 4.1 Skeleton Precision

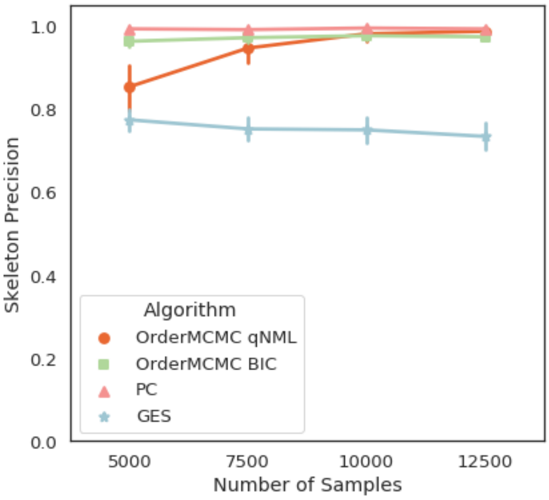


## 4.2 Skeleton Recall

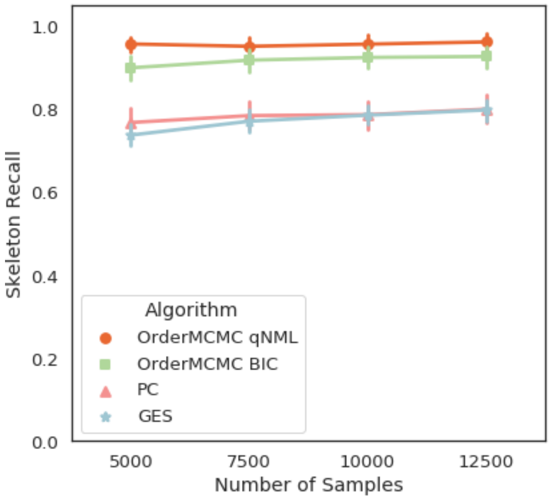


## 4.3 V-Structure Precision

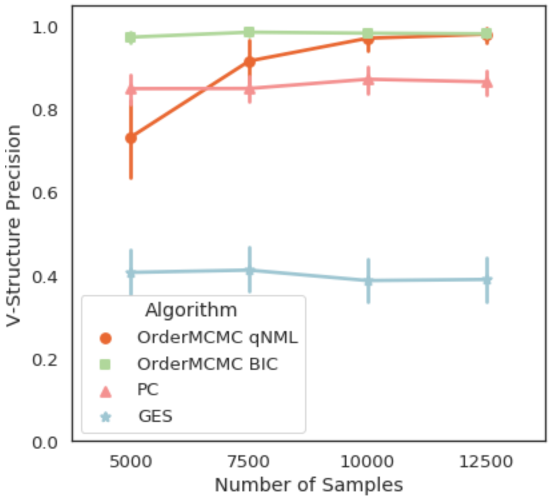

### 4.4 V-Structure Recall

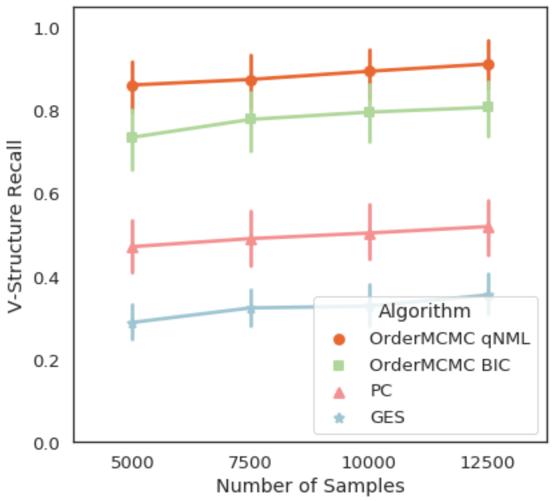


## 5 Improving with Less Variables

If the performance shown is this datasheet is not acceptable, improvements can generally be made by reducing the number of variables. The following plot show the expected performance gain using less variables for the Maternal Health in Uttar Pradesh Dataset:

### 5.1 Skeleton Precision

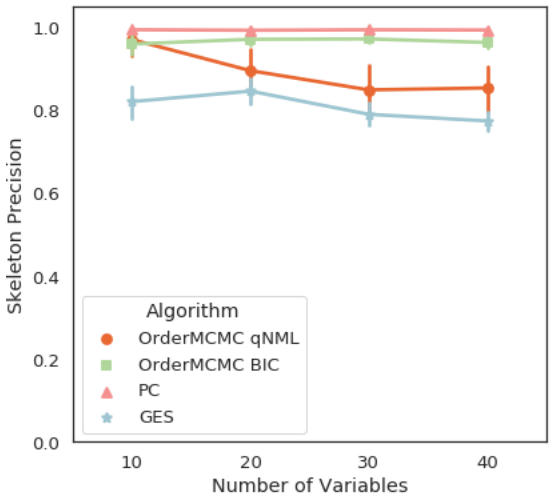


### 5.2 Skeleton Recall

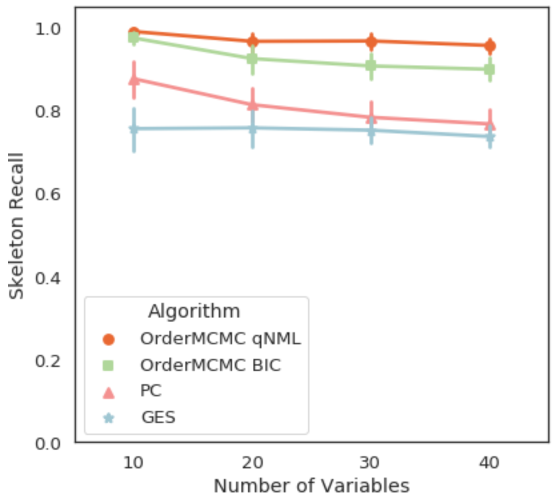


### 5.3 V-Structure Precision

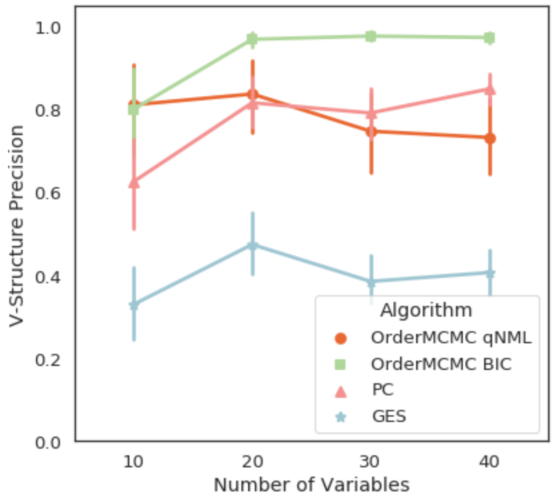


### 5.4 V-Structure Recall

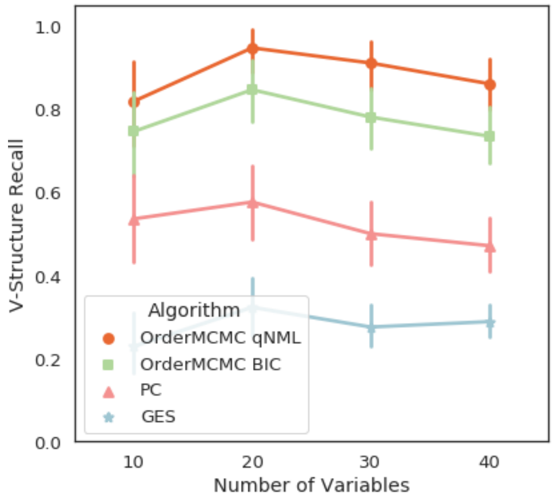

# (Supplementary Material C) Causal Datasheet for ALARM Dataset

Input Data Characteristics

**Validity Score:** 0.55 (0.15 std)

**Variables:** 40

**Samples:** 10,000

**Average Levels:** 3

**Alpha:** 6

**Maximum In-degree:** $\infty$

**Structure Types:** Forest Fire, IC-DAG, Barabasi-Albert, Waxman, Small World

## Recommendations

The following are a list of recommendations for using ALARM Dataset to learn a Bayesian network:

- Given the **0.80 PCOR threshold** supplied by the user, the ALARM Dataset **can** be used for learning a Bayesian network without further samples, constraints, or expert input.
- It is reccomended the **OrderMCMC** algorithm is used as this obtains the best performance.
- **PC** should be used if **skeleton precision** is the priority, while **OrderMCMC** should be used if **skeleton recall** is the priority
- **OrderMCMC** should be used if successfully capturing V-Structures is a priority

## 1 Correctness of Causal Effects

The **Proportion of Correct Odds Ratios**(PCOR) measures the proportion of interventional odds ratios a learnt BN correctly estimates.

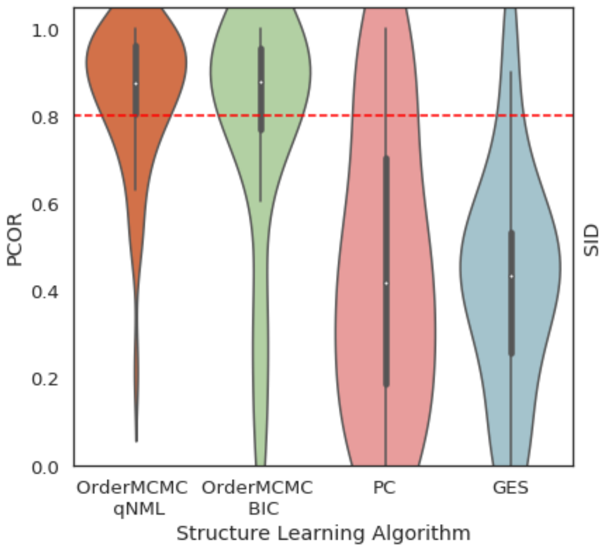


- Median PCOR using the **OrderMCMC (qNML)** algorithm = 0.88 (IQR = 0.16)
- Median PCOR using the **OrderMCMC (BIC)** algorithm = 0.88 (IQR = 0.19)
- Median PCOR using the **PC** algorithm = 0.42 (IQR = 0.52)
- Median PCOR using the **GES** algorithm = 0.43 (IQR = 0.28)

## 2 Learning the Skeleton

The following are plots showing what skeleton structure learning performance to expect given ALARM Dataset's properties:

### 2.1 Skeleton Precision

**Precision**, also known as the positive predictive value, is defined as the number of correct edge predictions made divided by all edge predictions made.

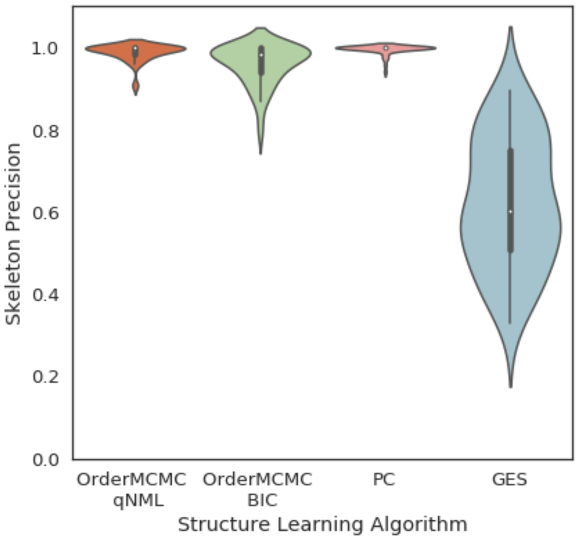


- Median skeleton precision using the **OrderMCMC (qNML)** algorithm = 1.0 (IQR = 0.02)
- Median skeleton precision using the **OrderMCMC (BIC)** algorithm = 0.98 (IQR = 0.06)
- Median skeleton precision using the **PC** algorithm = 1.0 (IQR = 0.0)
- Median skeleton precision using the **GES** algorithm = 0.6 (IQR = 0.24)

### 2.2 Skeleton Recall

**Recall** is the proportion of predicted positive cases over true positive cases.

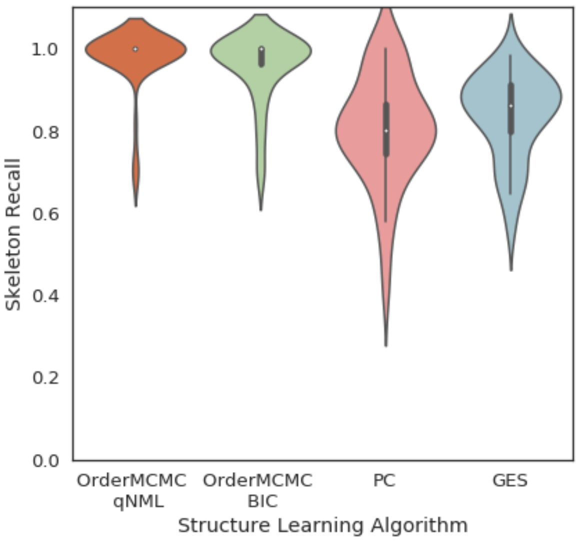


- Median skeleton recall using the **OrderMCMC (qNML)** algorithm = 1.0 (IQR = 0.0)
- Median skeleton recall using the **OrderMCMC (BIC)** algorithm = 1.0 (IQR = 0.04)
- Median skeleton recall using the **PC** algorithm = 0.8 (IQR = 0.12)
- Median skeleton recall using the **GES** algorithm = 0.86 (IQR = 0.11)

## 3 Learning the Direction

The following are plots showing what V-structure structure learning performance to expect given ALARM Dataset properties:

### 3.1 V-Structure Precision

The precision with respect to V-Structures is the proportion of learnt V-Structures which are present in the true CPDAG.

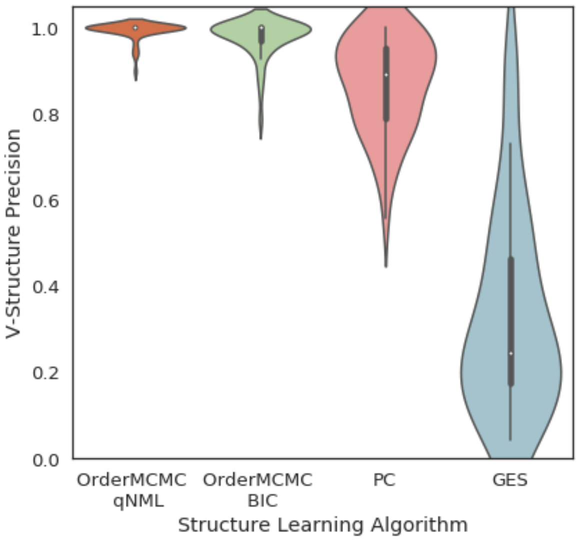


- Median V-Structure precision using the **OrderMCMC (qNML)** algorithm = 1.0 (IQR = 0.0)
- Median V-Structure precision using the **OrderMCMC (BIC)** algorithm = 1.0 (IQR = 0.03)
- Median V-Structure precision using the **PC** algorithm = 0.89 (IQR = 0.17)
- Median V-Structure precision using the **GES** algorithm = 0.24 (IQR = 0.29)

### 3.2 V-Structure Recall

Recall of V-Structures is the proportion of the true V-Structures that are present in the predicted case.

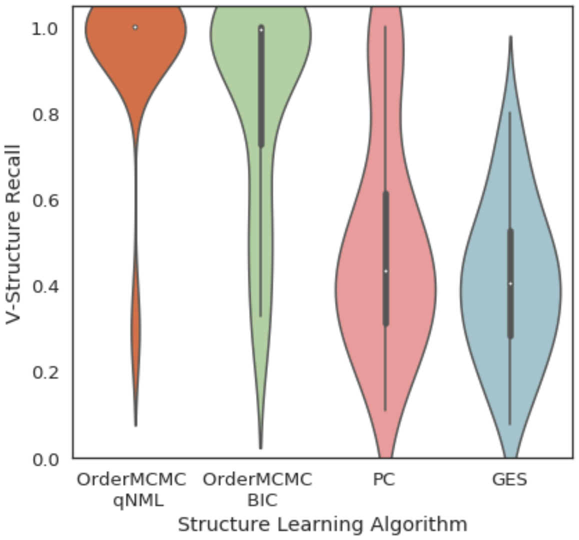


- Median V-Structure recall using the **OrderMCMC (qNML)** algorithm = 1.0 (IQR = 0.0)
- Median V-Structure recall using the **OrderMCMC (BIC)** algorithm = 0.99 (IQR = 0.27)
- Median V-Structure recall using the **PC** algorithm = 0.43 (IQR = 0.3)
- Median V-Structure recall using the **GES** algorithm = 0.4 (IQR = 0.24)

## 4 Improving with More Samples

If the performance shown is this datasheet is not acceptable, improvements can generally be made by adding more samples. The following plot shows expected performance gain with further samples for ALARM Dataset:

### 4.1 Skeleton Precision

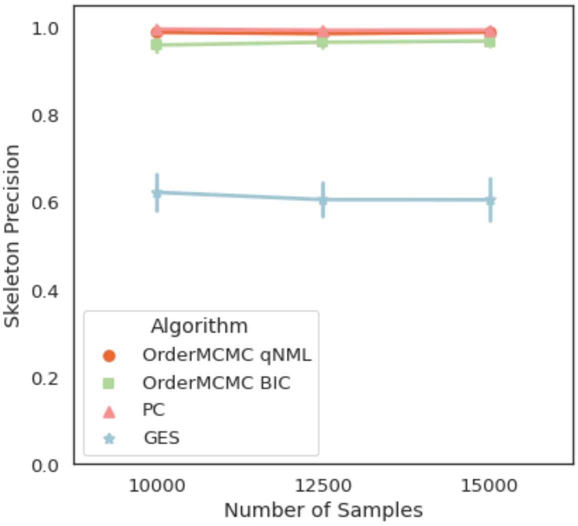


### 4.2 Skeleton Recall

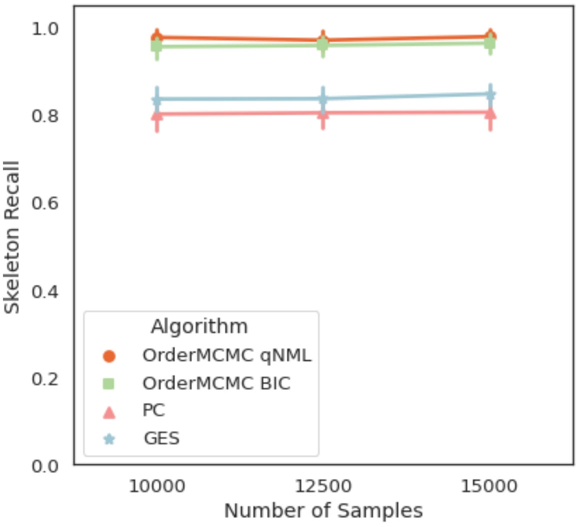


### 4.3 V-Structure Precision

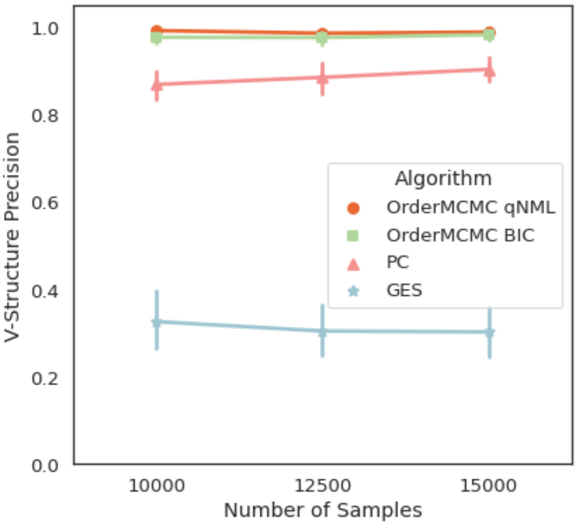


### 4.4 V-Structure Recall

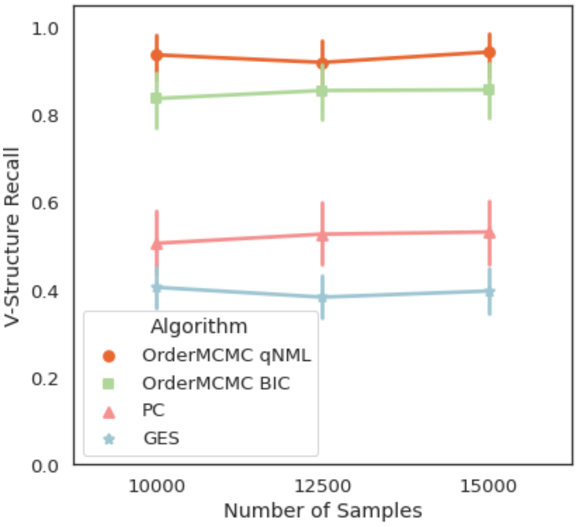


## 5 Improving with Fewer Variables

If the performance shown is this datasheet is not acceptable, improvements can generally be made by reducing the number of variables. The following plot show the expected performance gain using less variables for the ALARM Dataset:

### 5.1 Skeleton Precision

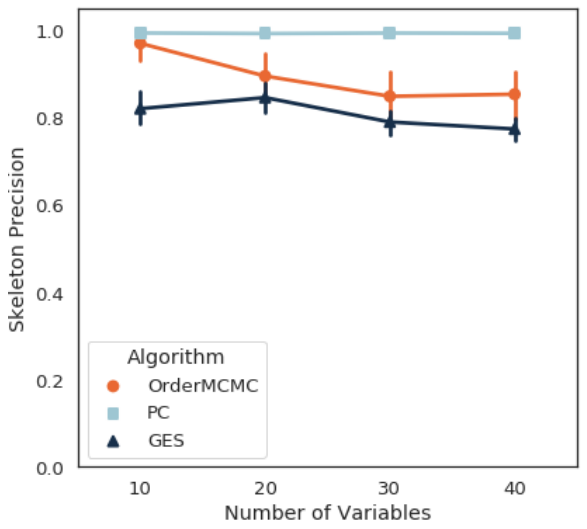


### 5.3 V-Structure Precision

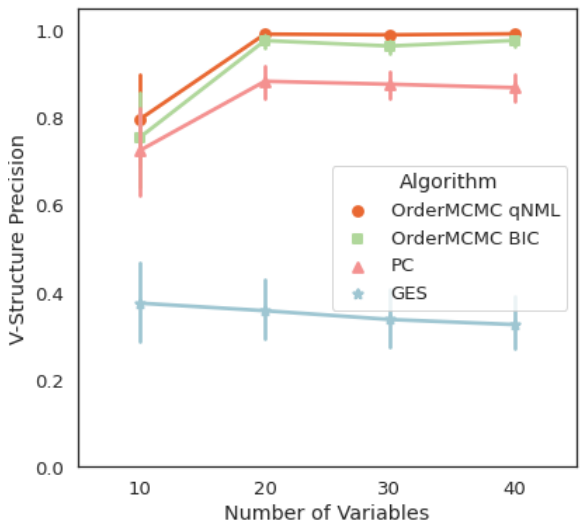


### 5.2 Skeleton Recall

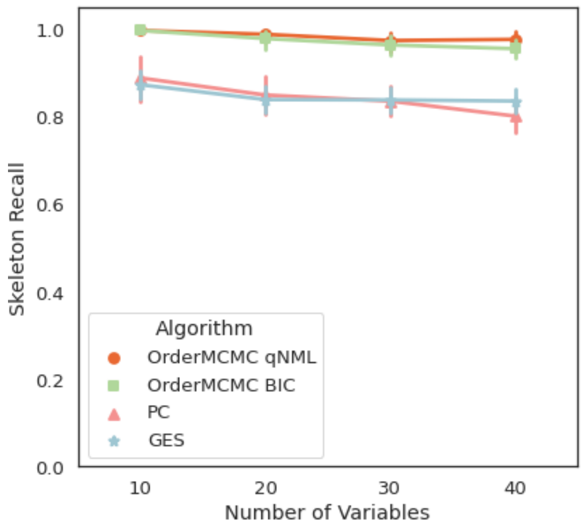


### 5.4 V-Structure Recall

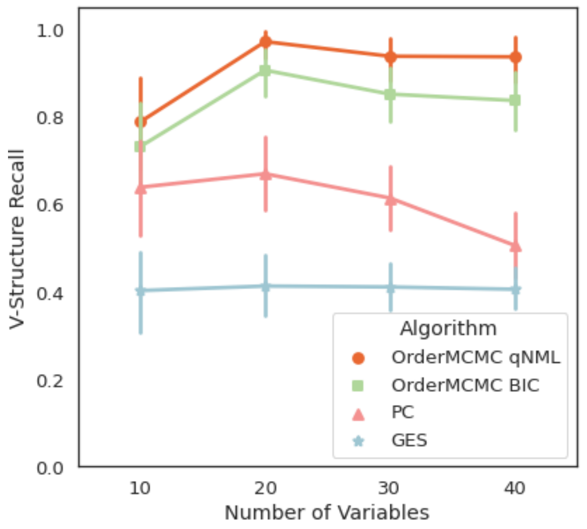

## Supplimentary Material D: Preliminary Results on Non-Uniform $\alpha$ Estimation and Meta-Feature Similarity

In this section we provide some insight into the work we are performing to resolve the limitations discussed in the main text, along with some initial results.

### Non-uniform $\alpha$ Estimation

We have observed in the ALARM dataset that we can overestimate performance when the parameters are non-uniform, it follows to improve the current performance, we should attempt to capture this case in our synthetic generation. In our preliminary test of this idea, we employ the following algorithm to estimate the non-uniform alpha:

**Algorithm 1:** Alpha-Estimation Synthetic Generation Scheme

Given:
- An Initial Estimate BN $\mathcal{B}$ of a real dataset
- A Synthetic DAG

1.For each real node:
a.Extract the corresponding parameters $\theta_i$
b.Stack this parameter estimate $t$ times, adding normally distributed noise $\mathcal{N}(0, v)$. *Both t and v are hyper-parameters which control the amount of regularization on the alpha estimates.*
c.Using Maximium Likelihood Estimation: Estimate alpha for each CPT in $\mathcal{B}$ (Huang, 2005)
2.For each Synthetic Node:
a.Assign each alpha to a synthetic node inversely proportionate to the in-degree, with some random noise.
b.Sample, using the assigned alpha, from a Dirichlet distribution to aquire the synthetic parameters

As described in the algorithm, this method requires an estimate DAG in order to function. How to best form this estimate, whether that be an agreement graph (as in the Intersection-validation paper (Viinikka et al., 2018)), high recall, or high precision DAG, is future work. In the following experiments we provide the oracle BN to the algorithm.

Figure S1 shows the comparison between the CDG-T estimates and the real ALARM performance. There is generally a closer alignment compared to the estimates formed using a uniform alpha value, particularly for V-Structure recall (Figure S1d) which no-longer estimates performance with a median of 1.

To further test the alpha estimation we use another expert-designed dataset with a known-ground truth – Insurance (Binder et al., 1997). Because Insurance is a dataset designed to capture the probability of car theft, it contains events which are very low frequency. Much like ALARM, some CPTs of the Insurance dataset have a high marginal imbalance. Therefore, this dataset suits our purposes as another test case for our alpha estimate technique. Compared to synthetic datasets which assume uniform alpha, there is overall much better alignment when we use the alpha estimation technique, bringing both skeleton and V-structure estimates further into alignment with the ground truth (Figure S2. Results from the uniform estimates can be found in the supplementary material).

Finally, we test our alpha estimation algorithm on another expert-designed BN – Child (Dawid, 1992). This data set does not have any notable characteristics in its CPTs, and could generally be generated using Dirichlet distributions with a uniform alpha. As anticipated, our calculated performance estimates for this dataset using the uniform alpha and estimated alpha have minimal differences (Figure S3).

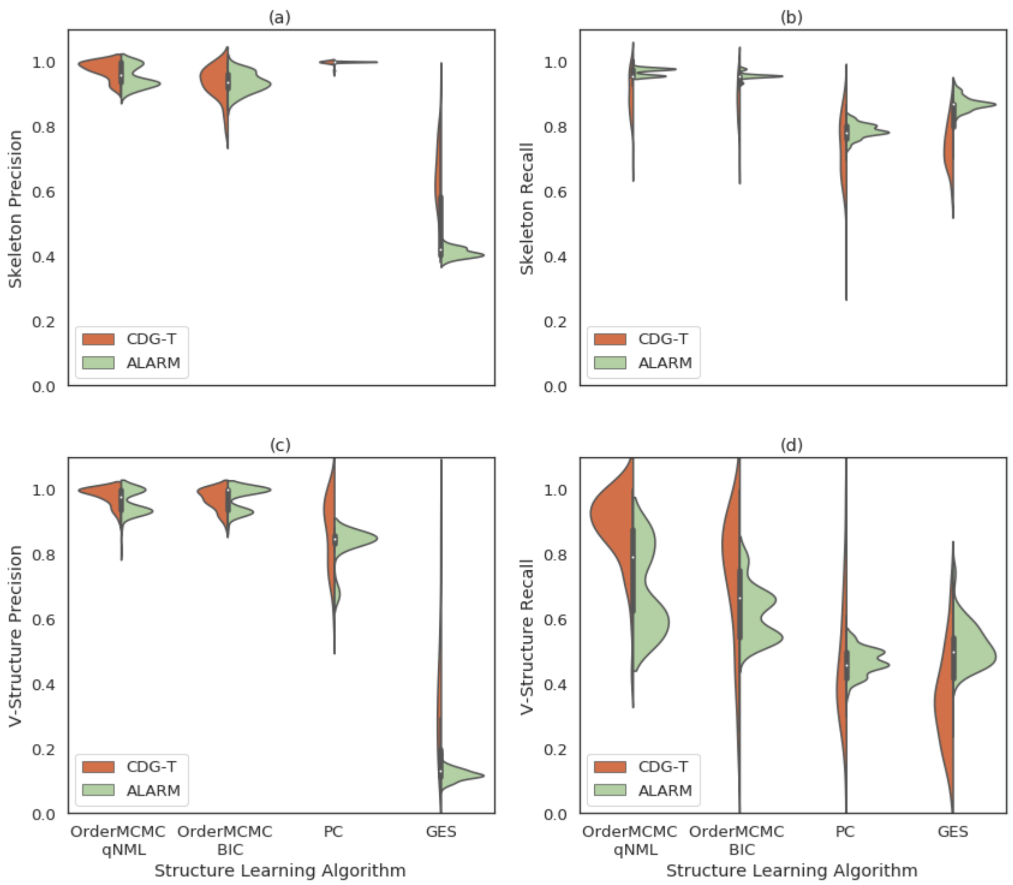


Figure S1: The performance obtained on CDG-T datasets using the alpha estimation technique vs. the ALARM dataset over 100 runs. **(a)**: Skeleton Precision; **(b)**: Skeleton Recall; **(c)**: V-structure Precision; **(d)**: V-structure Recall. General alignment between estimates and actual performance, with skeleton recall now slightly underestimated. V-Structure Recall estimates now aligns better with actual performance.

All of the expert-designed BNs can be found in the BNlearn repository: https://www.bnlearn.com/bnrepository/ (Scutari, 2009).

## Meta-feature Similarity Score

In presenting performance estimates from synthetic data, we assume that they are similar enough to a given real dataset to be valid. Because a practitioner must specify some set of unobservable BN characteristics, further guidance on whether these selections were reasonable is desirable.

We borrow the concept of defining meta-features and computing dataset similarities from the Bayesian optimization hyperparameter initialization literature (Wistuba et al., 2015). Currently, because our intended datasets are categorical, a small set of information theoretic meta-features (i.e., Shannon's entropy and Concentration Coefficient) are computed for both the real and synthetic datasets(Michie et al., 1994; Alexandros and Melanie, 2001). Similarity between the synthetic and real datasets can then be assessed via a meta-feature similarity score, which is simply the mean cosine similarity between the meta-features of the real and synthetic datasets. The formula is as follows:

$$\text{MF-Similarity}(R, S) = \frac{1}{|S|} \sum_{i}^{|S|} \frac{M(R) \cdot M(S_i)}{\|M(R)\| \times \|M(S_i)\|}$$

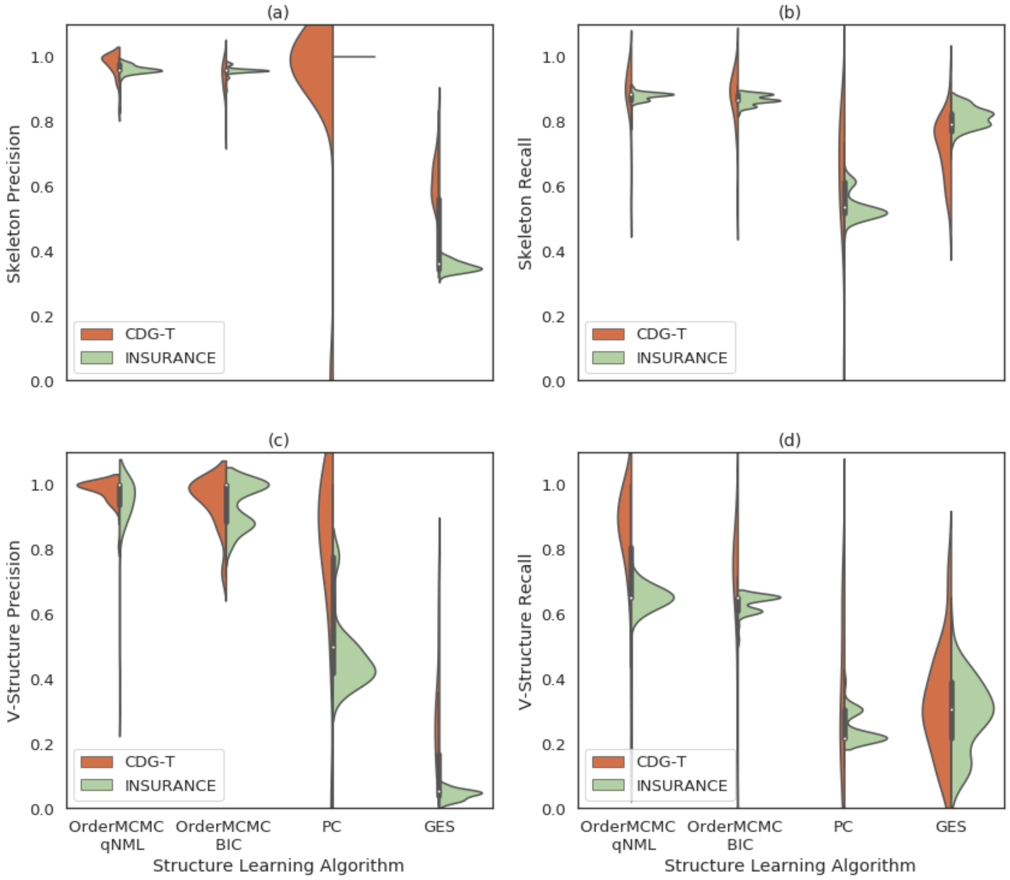


Figure S2: The performance obtained on CDG-T datasets using the alpha estimation technique vs. the Insurance dataset over 100 runs. **(a)**: Skeleton Precision; **(b)**: Skeleton Recall; **(c)**: V-structure Precision; **(d)**: V-structure Recall. Estimates for OrderMCMC generally correct, with slight overestimation for V-Structure Recall. V-Structure precision is overestimated for PC and GES, with skeleton precision for GES also being overestimated.

where $M$ is a function computing the meta-features for a dataset, $R$ is a real dataset, and $S$ is the set of synthetic datasets. Each meta-feature is computed as the set $\{$min, median, max$\}$ over the variables. Careful selection of meta-features is crucial in order to obtain a reliable score. One should avoid selecting meta-features with any overlap to the user-specified properties (e.g., sample size, number of variables). As a gut check, we computed the meta-feature similarity for the datasets generated assuming an alpha using the non-uniform alpha estimates, as well as the datasets generated assuming a uniform alpha vector for some common datasets(Beinlich et al., 1989; Binder et al., 1997; Dawid, 1992) and the Surgo Household dataset (Table S1 using the PyMFE library(Alcobaça et al., 2020)). As expected, synthetic datasets are more similar to the source datasets when non-uniform alpha estimates are used. While there appears to be a ceiling effect, we hope this provides a basis by which estimates in a causal datasheet can be used practically.

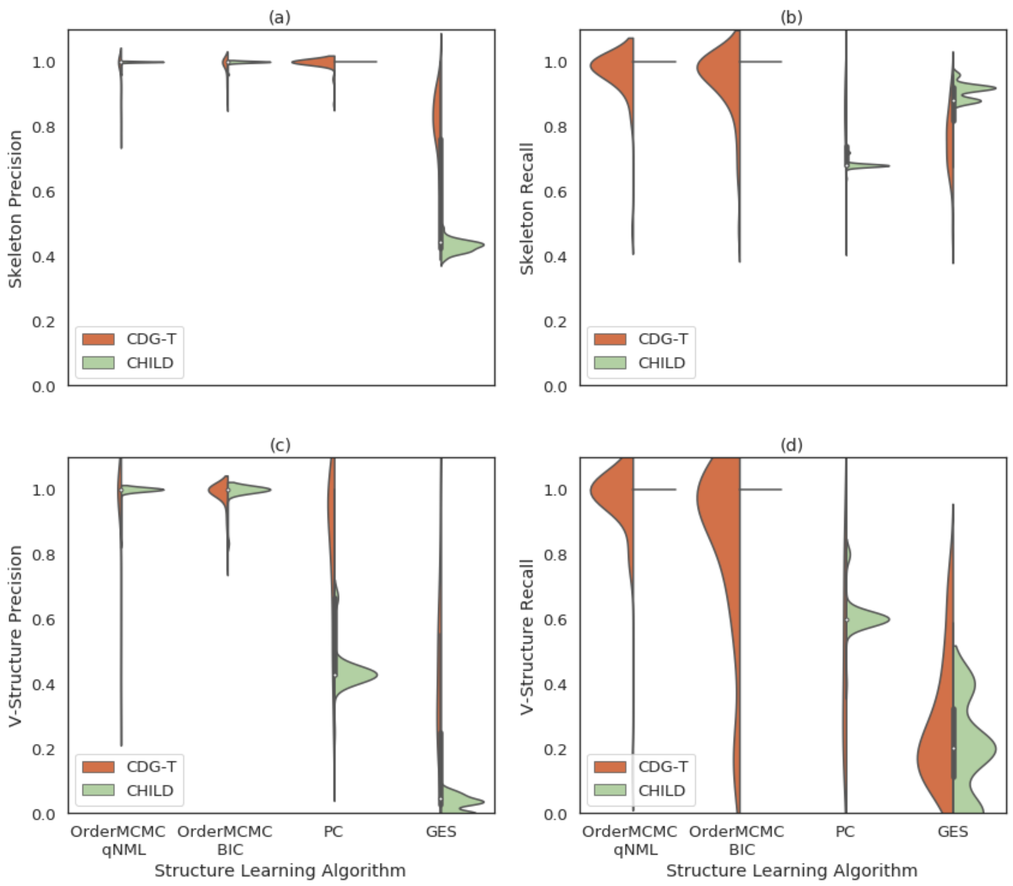


Figure S3: The performance obtained on CDG-T datasets using the alpha estimation technique vs. the Child dataset over 100 runs. (**a**): Skeleton Precision; (**b**): Skeleton Recall; (**c**): V-structure Precision; (**d**): V-structure Recall. General alignment for the OrderMCMC algorithm. Overestimation for GES precision, with wide performance margins for GES and PC V-Structure performance.

| Dataset | Uniform Alpha | Non-Uniform Alpha |
|---|---|---|
| Surgo Household | .89 | .96 |
| ALARM | .91 | .99 |
| Insurance | .89 | .99 |
| Child | .97 | .98 |

**Table S1.** Meta-feature similarity scores for each dataset using different parameter generation schemes. The estimate DAG provided to the alpha estimation scheme for the Surgo Household dataset was learnt using the OrderMCMC + qNML algorithm.

## SUPPLIMENTARY MATERIAL E: UNIFORM ALPHA

In this section we provide the results for the insurance and child datasets using a uniform alpha. These can be compared to the results in the previous section where a non-uniform alpha estimates was used.

### Insurance dataset

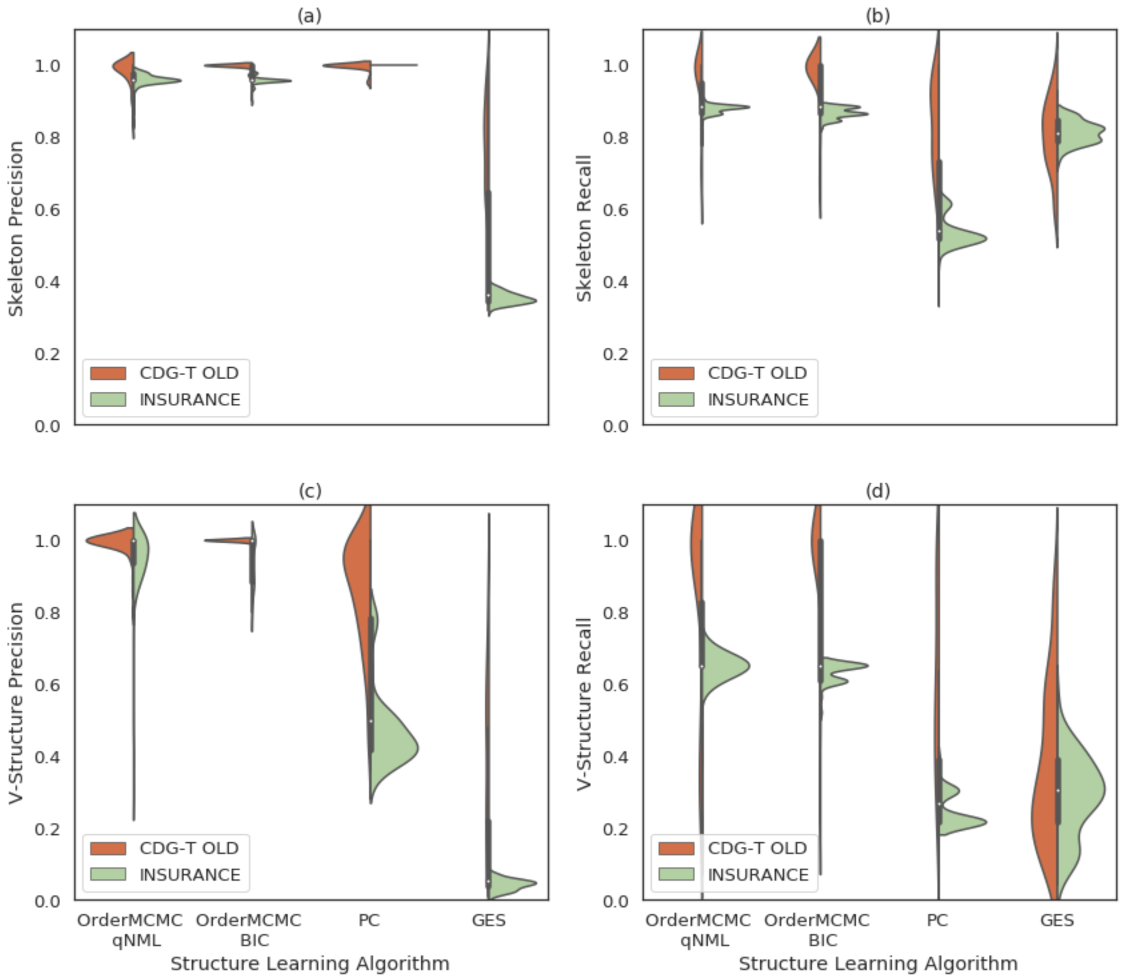


Figure S4: The performance alignment for CDG-T and Insurance using Uniform alpha estimates.

## Child dataset

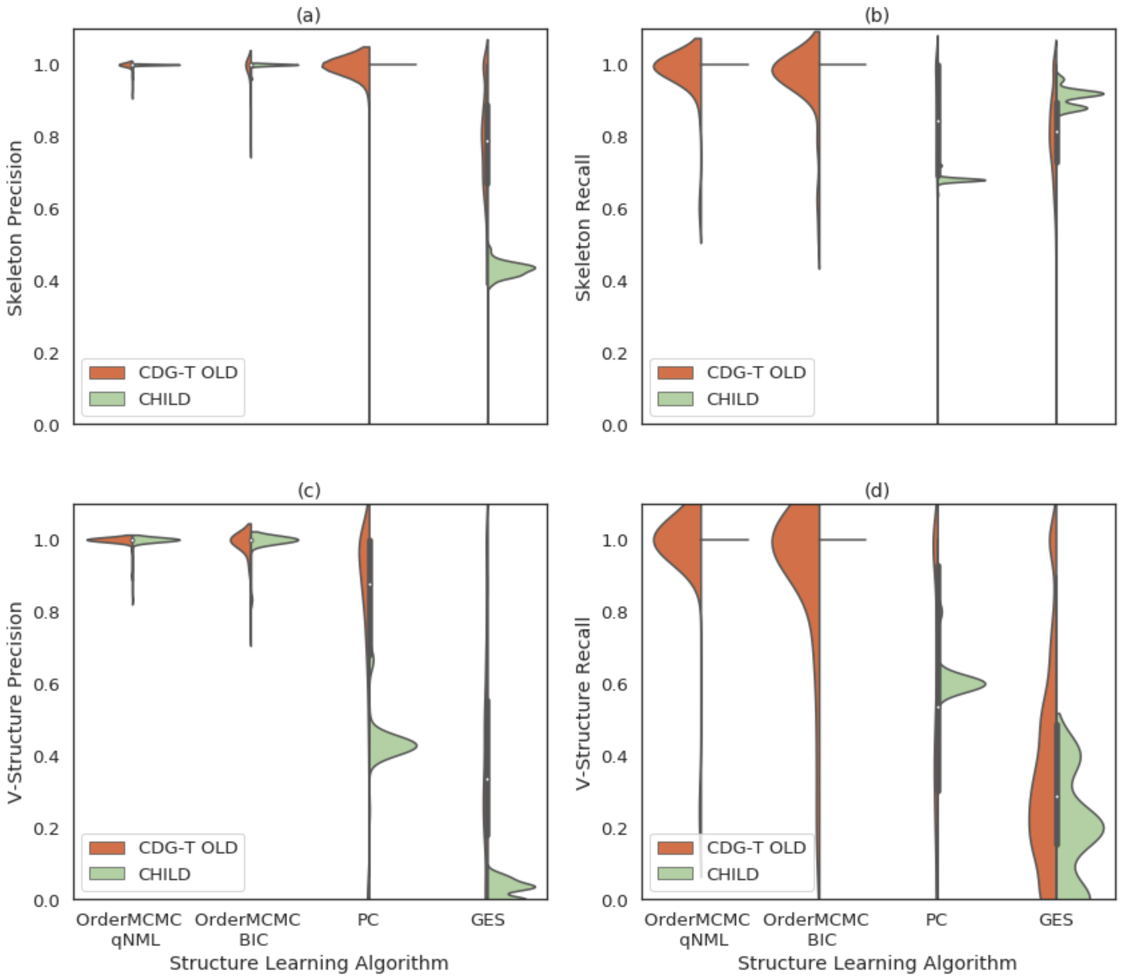


Figure S5: The performance alignment for CDG-T and child using Uniform alpha estimates.

## REFERENCES

Alcobaça, E., Siqueira, F., Rivolli, A., Garcia, L. P., Oliva, J. T., de Carvalho, A. C., et al. (2020). Mfe: Towards reproducible meta-feature extraction. *Journal of Machine Learning Research* 21, 1–5

Alexandros, K. and Melanie, H. (2001). Model selection via meta-learning: a comparative study. *International Journal on Artificial Intelligence Tools* 10, 525–554

Beinlich, I. A., Suermondt, H. J., Chavez, R. M., and Cooper, G. F. (1989). The alarm monitoring system: A case study with two probabilistic inference techniques for belief networks. In *AIME 89* (Springer). 247–256

Binder, J., Koller, D., Russell, S., and Kanazawa, K. (1997). Adaptive probabilistic networks with hidden variables. *Machine Learning* 29, 213–244

Dawid, A. P. (1992). Prequential analysis, stochastic complexity and bayesian inference. *Bayesian statistics* 4, 109–125

Huang, J. (2005). Maximum likelihood estimation of dirichlet distribution parameters. *CMU Technique Report*

Michie, D., Spiegelhalter, D. J., Taylor, C., et al. (1994). Machine learning. *Neural and Statistical Classification* 13, 1–298

Scutari, M. (2009). Learning bayesian networks with the bnlearn r package. *arXiv preprint arXiv:0908.3817*

Viinikka, J., Eggeling, R., and Koivisto, M. (2018). Intersection-validation: A method for evaluating structure learning without ground truth. In *Proceedings of the Twenty-First International Conference on Artificial Intelligence and Statistics*, eds. A. Storkey and F. Perez-Cruz (Playa Blanca, Lanzarote, Canary Islands: PMLR), vol. 84 of *Proceedings of Machine Learning Research*, 1570–1578

Wistuba, M., Schilling, N., and Schmidt-Thieme, L. (2015). Learning data set similarities for hyperparameter optimization initializations. In *Metasel@ pkdd/ecml*. 15–26
